# Functional Aquafeeds for Climate-Resilient Finfish Culture: A PRISMA-Guided Systematic Evidence Map of Nutritional Strategies for Thermal-Stress Tolerance, Immunity and Metabolic Homeostasis

**DOI:** 10.3390/vetsci13080756

**Published:** 2026-07-30

**Authors:** Md Hashibur Rahman, Hyuncheol Jeon, Haham Kim, Seunghyung Lee

**Affiliations:** 1 Major of Aquaculture and Applied Life Sciences, Division of Fisheries Life Sciences, Pukyong National University, Busan 48547, Republic of Korea; hashibur@pukyong.ac.kr (M.H.R.); conjp@naver.com (H.J.); haham7@naver.com (H.K.); 2 Interdisciplinary Program of Marine and Fisheries Sciences and Convergent Technology, Pukyong National University, Busan 48513, Republic of Korea; 3 Feeds and Foods Nutrition Research Center, Pukyong National University, Busan 48547, Republic of Korea

**Keywords:** climate-resilient aquafeeds, thermal stress, functional feed additives, antioxidant defense, fish immunity, metabolic homeostasis, PRISMA evidence map

## Abstract

Thermal variability associated with climate change is an increasing constraint for finfish aquaculture because water temperature directly affects feed intake, nutrient utilization, endocrine stress responses, oxidative balance, immunity, intestinal integrity and survival. The literature search was conducted without a lower publication-year restriction through 5 July 2026. Database and citation-linked searches identified 386 candidate records, of which 274 remained after duplicate removal; 71 full-text articles were assessed; and 28 peer-reviewed primary feeding studies with publicly available final versions of record were retained for qualitative evidence mapping. The eligible studies were published between 2012 and 2026, with 2012 representing the earliest eligible publication rather than a prespecified starting year. Functional aquafeeds have therefore emerged as promising nutritional tools for supporting physiological resilience under heat, cold and sub-optimal temperature conditions. This review developed a PRISMA-guided systematic evidence map of dietary functional-aquafeed strategies evaluated for thermal-stress tolerance, immune regulation, antioxidant defense and metabolic homeostasis in cultured finfish. The main synthesis emphasizes studies with direct dietary thermal-stress interventions and extractable mechanistic outcomes, including sodium butyrate, organic selenium, selenium nanoparticles with riboflavin, astaxanthin, *Bacillus* spp., propolis, bay laurel essential oil, *Spirulina*-based strategies and essential oils. Overall, the available evidence suggests that selected functional aquafeeds may support thermal-stress resilience through coordinated improvements in antioxidant capacity, reduced oxidative damage, modulation of cortisol/glucose and heat-shock responses, enhancement of innate and mucosal immunity, improved intestinal or tissue condition and stabilization of metabolic indicators. The most biologically coherent translational patterns were observed when dietary interventions improved multiple response domains simultaneously rather than a single biomarker. However, such cross-domain improvement was interpreted as mechanistic and translational support rather than as an independent indicator of evidence confidence, which depended primarily on independent replication, consistency of response direction and methodological quality. Nevertheless, the current evidence remains uneven across species, stress models, life stages and outcome domains, with limited data for marine carnivores, repeated temperature fluctuation, post-stress recovery, disease challenge and multiomics integration. Therefore, the findings should be interpreted as a structured evidence map rather than universal formulation guidance. Future studies should use standardized thermal-challenge protocols, dose–response designs, clear tank-level experimental-unit reporting and integrated biomarker panels to support practical development of climate-resilient functional aquafeeds.

## Simple Summary

Farmed fish are increasingly exposed to stressful water temperatures as climate patterns become less predictable. Nutrition offers a practical way to help fish cope with these conditions because some feed ingredients can strengthen natural defense systems, protect the intestine and support recovery after heat or cold exposure. This systematic review organized feeding-trial evidence on functional aquafeeds tested in finfish under temperature-related challenges. The reviewed studies indicate that ingredients such as sodium butyrate, selenium sources, astaxanthin, probiotics, plant extracts, essential oils and algae-based products may be useful when they improve several biological responses together rather than one marker alone. The most relevant effects involved better antioxidant protection, immune balance, intestinal condition, stress regulation, energy use and survival. The systematic review also shows that the evidence is still uneven among species and study designs. More standardized trials are needed, especially in marine fish and recovery or disease-challenge models, to support climate-ready aquafeed development.

## 1. Introduction

Aquaculture has become a central pillar of global aquatic food production, nutrition security and rural livelihoods, particularly as capture fisheries remain constrained by biological limits, stock depletion and climate-related uncertainty [1,2,3,4]. The most recent global assessments indicate that aquaculture is no longer a supplementary source of aquatic food but a dominant contributor to human fish supply, with aquaculture production of aquatic animals surpassing capture fisheries production for the first time in 2022 [1]. This transition has intensified the need for production systems that are not only efficient and economically viable but also resilient to environmental instability, disease outbreaks and resource constraints [2,5,6]. Within this context, aquafeed nutrition has shifted from a traditional emphasis on growth maximization and feed conversion toward a broader concept of nutritional resilience, in which diets are formulated to support physiological robustness, immune competence, oxidative balance and metabolic flexibility under changing culture conditions [7,8,9,10].

Climate change is emerging as one of the most important external pressures on aquaculture, particularly through ocean and freshwater warming, increasing frequency of marine and inland heatwaves, seasonal temperature instability, altered oxygen availability and changes in pathogen ecology [5,6,11,12,13]. Because fish are ectothermic animals, water temperature directly influences biochemical reaction rates, aerobic scope, feed intake, digestion, nutrient absorption, endocrine stress responses, immune function and energy allocation [14,15,16,17,18]. Thermal conditions outside the optimal range can impair growth and feed efficiency by redirecting energy from somatic deposition toward maintenance, osmoregulation, stress-response pathways and cellular repair [15,19,20]. Both acute and chronic temperature stress may also compromise welfare and production by increasing cortisol and glucose mobilization, disturbing lipid and amino acid metabolism, altering hematological and plasma biochemical profiles, damaging intestinal integrity and increasing susceptibility to opportunistic pathogens [20,21,22,23,24].

At the cellular level, thermal stress commonly induces oxidative stress, protein damage, inflammatory imbalance and metabolic reprogramming [20,22,23,24,25,26]. Elevated temperature can increase mitochondrial respiration and reactive oxygen species production, whereas cold stress may reduce enzyme efficiency, alter membrane fluidity and disrupt energy supply–demand balance [18,25,27]. Fish respond through coordinated activation of heat-shock proteins, antioxidant enzymes and endocrine stress pathways, including HSP70/HSP90 expression, superoxide dismutase, catalase, glutathione peroxidase, glutathione-related enzymes, cortisol-mediated gluconeogenesis and altered plasma metabolites such as glucose, cholesterol, triglycerides and total protein [20,21,22,24,28]. However, when these responses are insufficient or prolonged, thermal stress may result in lipid peroxidation, immune suppression, intestinal dysbiosis, apoptosis, impaired nutrient utilization and reduced survival [20,24,25,29,30]. Therefore, maintaining immune, antioxidant and metabolic homeostasis under thermal stress has become a key objective for climate-resilient finfish culture.

Functional aquafeeds offer a practical and non-pharmaceutical strategy for improving fish resilience to temperature-related stressors [7,9,10,31,32]. Unlike conventional feeds designed mainly to satisfy basal nutrient requirements, functional aquafeeds include nutrients, bioactive compounds or microbial modulators that can enhance physiological performance beyond normal growth, particularly by supporting gut barrier function, antioxidant capacity, innate immunity, stress tolerance and disease resistance [8,31,33,34]. Potential functional strategies for thermal-stress resilience include antioxidant micronutrients such as vitamin C, vitamin E, selenium, zinc and copper; functional amino acids such as taurine, arginine, glutamine, methionine and γ-aminobutyric acid; microbial additives such as probiotics, prebiotics, synbiotics and postbiotics; phytogenic compounds including essential oils, polyphenols and plant extracts; and marine- or insect-derived bioactives such as seaweed polysaccharides, chitin, chitosan and nucleotides [10,32,34,35,36,37].

The biological plausibility of functional aquafeeds for thermal-stress mitigation is supported by several mechanistic pathways. Antioxidant nutrients and phytogenic compounds may reduce oxidative damage by strengthening enzymatic and non-enzymatic antioxidant systems, stabilizing membranes and modulating nuclear factor erythroid 2-related factor 2-associated antioxidant signaling [25,35,38]. Functional amino acids can influence protein synthesis, osmoregulation, bile acid conjugation, neurotransmission, nitric oxide production, glutathione metabolism and immune-cell function, thereby linking nutrient metabolism with stress adaptation [10,37,39]. Probiotics, prebiotics and synbiotics may improve resilience by stabilizing gut microbiota, enhancing short-chain fatty acid production, improving intestinal morphology, regulating mucosal immunity and reducing pathogen colonization under stress-challenged conditions [32,33,34,40]. Similarly, phytobiotics and marine polysaccharides may provide antioxidant, antimicrobial, anti-inflammatory and immunostimulatory effects through modulation of cytokines, complement activity, lysozyme activity, mucin production and tight-junction integrity [31,38]. These mechanisms suggest that dietary interventions can influence thermal tolerance not through a single pathway but through integrated immune–metabolic regulation.

Despite the rapid expansion of functional feed research in aquaculture, the evidence base remains fragmented across fish species, stress models, functional-ingredient classes, feeding durations, temperature-challenge designs, tissues and measured endpoints [7,9,10,20]. Many studies report growth, survival, feed utilization or antioxidant enzyme activities, whereas others focus on plasma metabolites, cytokine expression, heat-shock proteins, intestinal morphology, gut microbiota or transcriptomic/metabolomic profiles [20,28,37,41]. This heterogeneity makes direct quantitative comparison difficult but also creates an opportunity for systematic evidence mapping. In particular, there is a need to identify which nutritional strategies have been tested most frequently, which cultured finfish species and life stages are over- or under-represented, which immune and metabolic biomarkers are consistently used, and which response patterns are associated with improved tolerance to heat stress, cold stress or temperature fluctuation. A structured synthesis is also needed to distinguish between general health-promoting effects under normal conditions and specific resilience-enhancing effects under thermal challenge.

Previous reviews have addressed fish immunonutrition, functional feed additives, probiotics and prebiotics, nutrigenomics, oxidative stress or climate-related thermal stress separately [7,8,9,10,20,32,34,41]. However, fewer studies have systematically integrated these areas around the central question of climate-resilient aquafeed design. In particular, the current literature lacks a PRISMA-guided evidence map that links functional feed strategies with thermal-stress tolerance, immune responses, antioxidant defense and metabolic homeostasis in cultured finfish. Such an approach is important because thermal-stress resilience is not determined only by survival at extreme temperatures but also by the capacity to maintain feed intake, nutrient utilization, redox balance, mucosal integrity, immune competence and metabolic stability during and after thermal disturbance [10,15,17,20].

Therefore, this review aimed to systematically map the evidence on functional aquafeeds for climate-resilient finfish culture, with particular emphasis on nutritional strategies for thermal-stress tolerance, immunity and metabolic homeostasis. Specifically, the review aimed to: (i) identify the major functional-ingredient classes evaluated under heat stress, cold stress or temperature fluctuation; (ii) map the cultured finfish species, life stages, feeding durations and thermal-challenge protocols used in the literature; (iii) synthesize reported effects on growth, survival, feed utilization, antioxidant defense, immune responses, stress biomarkers, gut health and metabolic indicators; and (iv) highlight research gaps and methodological limitations that currently restrict translation into practical feed formulation. By applying a PRISMA-guided systematic evidence map, this review seeks to provide a transparent foundation for designing functional aquafeeds that support climate resilience in cultured finfish.

## 2. Materials and Methods

### 2.1. Review Design and Reporting Framework

This manuscript was developed as a PRISMA-guided systematic evidence map to identify, classify and synthesize the available evidence on functional aquafeeds for improving thermal-stress resilience in cultured finfish. This review was performed in accordance with the PRISMA (Preferred Reporting Items for Systematic Reviews and Meta-Analyses) guidelines. The review focused on dietary nutritional strategies associated with thermal-stress tolerance, immune regulation, antioxidant defense and metabolic homeostasis. The methodology followed the Preferred Reporting Items for Systematic Reviews and Meta-Analyses 2020 statement to ensure transparent reporting of study identification, screening, eligibility assessment and synthesis [42]. The literature search was reported with reference to the PRISMA-S extension to improve reproducibility of the search strategy [43]. Because the included studies were expected to differ in fish species, functional-ingredient classes, thermal-challenge protocols, feeding duration and measured endpoints, the primary synthesis was designed as a systematic evidence map. Where quantitative pooling was not appropriate, the narrative synthesis was guided by the Synthesis Without Meta-analysis reporting guideline [44].

The review addressed the following research question: which dietary functional-aquafeed strategies have been evaluated for improving thermal-stress resilience in cultured finfish, and which immune, antioxidant and metabolic responses are most consistently associated with improved tolerance? The review protocol information was retrospectively registered on the Open Science Framework (OSF) for transparency. The eligibility criteria, search strategy, outcomes of interest, extraction categories, and synthesis approach were predefined before study screening. Registration details: [registration ID: q5csy; registry URL: https://doi.org/10.17605/OSF.IO/PVG4H].

**Figure 1:**
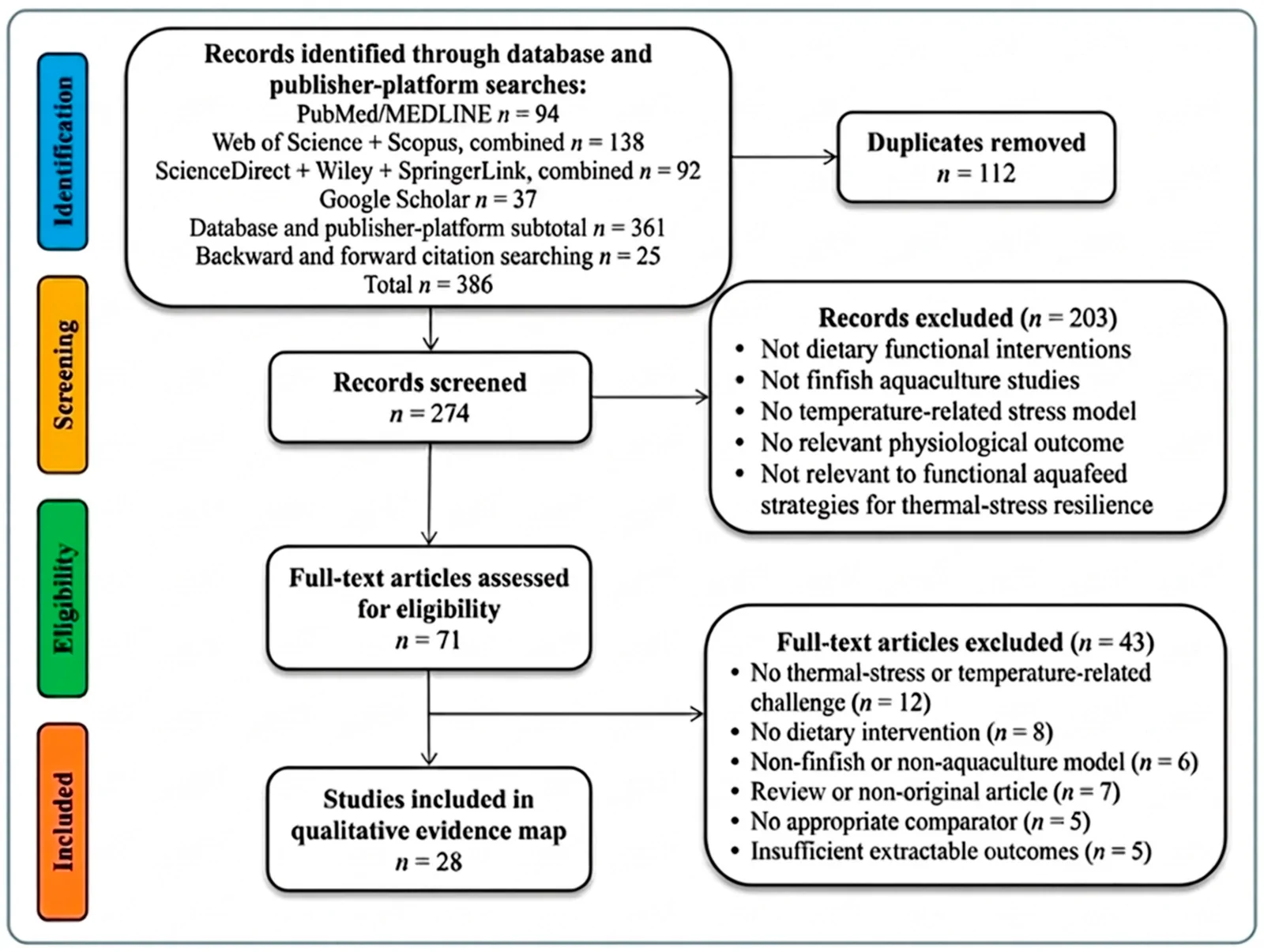
The PRISMA-guided study selection process for the systematic evidence map. The expanded database, publisher and citation-linked searches identified 386 candidate records. After removal of 112 duplicates, 274 records were screened by title and abstract, and 71 full-text articles were assessed for eligibility. Forty-three full-text articles were excluded because they lacked a thermal-stress component, dietary intervention, finfish model, appropriate comparator, original feeding-trial design or extractable relevant outcomes. Finally, 28 primary feeding studies were retained for qualitative evidence mapping of functional-aquafeed strategies under thermal-stress conditions in cultured finfish.

The complete evidence identification, screening, eligibility and inclusion process is presented in the PRISMA flow diagram (Figure 1), providing a transparent overview of how records were identified, screened, assessed for eligibility and retained for the final qualitative evidence map.

### 2.2. Eligibility Criteria

Eligibility criteria were structured according to a modified PICOS framework, including population, intervention, comparator, outcomes and study design. Studies were eligible when they used live freshwater or marine finfish species relevant to aquaculture production and evaluated them in a controlled dietary feeding trial. Eligible life stages included larvae, juveniles, grow-out fish and broodstock. Studies using aquatic invertebrates, including shrimp, crabs and mollusks, laboratory model fish such as zebrafish or medaka, ornamental fish without food-aquaculture relevance, cell-culture models, isolated tissues or ex vivo preparations were excluded.

The intervention of interest was dietary administration of a deliberately manipulated feed ingredient, nutrient, additive or formulation evaluated for a functional role during thermal stress. In this review, functional aquafeeds were defined as diets containing nutrients, additives or bioactive compounds intended to improve physiological function beyond basal nutrient supply, particularly stress tolerance, immune competence, antioxidant capacity, gut health or metabolic regulation. The term “functional” described the experimental purpose and use of the intervention rather than its essential or non-essential nutritional status. An intervention was considered functional only when: (i) it was intentionally varied as a dietary treatment against an appropriate basal, non-supplemented or lower-dose comparator; (ii) the study evaluated effects beyond routine provision of basal nutrient requirements or correction of a documented nutrient deficiency; and (iii) the intervention was assessed in relation to at least one predefined thermal-stress-resilience domain, including stress tolerance, antioxidant defense, immune regulation, gut or tissue integrity, metabolic stability, growth or survival under thermal challenge. Nutrients included only as normal components of a balanced diet, vitamin or mineral premix, or requirement-level formulation were not treated as functional interventions. Essential nutrients were eligible only when they were specifically tested as supplemental, graded or strategically formulated dietary treatments for thermal-stress resilience and when the comparator permitted their effects to be distinguished from basal nutrient provision.

Eligible interventions included functional amino acids and related compounds, vitamins, minerals and trace elements meeting the above functional-use criteria, phytogenic additives, microbial-based additives, marine- or algal-derived bioactives, insect-derived bioactive fractions, chitin, chitosan, nucleotides and other dietary compounds with clearly stated functional roles. Studies using injection, immersion, bath exposure or waterborne treatment without dietary administration were excluded because the review focused specifically on feed-based nutritional strategies. In studies containing both dietary and non-dietary treatment arms, only the eligible dietary arms were included in the evidence synthesis.

Eligible studies were required to include an appropriate control or comparator group, such as a basal diet, non-supplemented diet, commercial control diet, thermal-stress control or lower-dose dietary treatment. Studies without a clearly defined control group were excluded from the main synthesis. Eligible studies were also required to include a thermal-stress component. Thermal stress was defined as exposure to water temperature outside the optimal rearing range of the species or as an experimental temperature challenge designed to induce heat stress, cold stress or temperature fluctuation. Acute heat stress, chronic high-temperature exposure, acute cold stress, chronic low-temperature exposure, rapid temperature change, diel temperature fluctuation and seasonal temperature-challenge models were considered eligible when the dietary intervention was evaluated in relation to thermal-stress responses.

Studies were included when they reported at least one outcome related to thermal-stress resilience, immune response, antioxidant defense, metabolic homeostasis or aquaculture performance. Relevant outcomes included growth performance, feed utilization, survival, cortisol, glucose, lactate, heat-shock proteins, antioxidant enzymes, oxidative-damage markers, lysozyme activity, complement activity, immunoglobulin levels, cytokine expression, antimicrobial peptides, plasma metabolites, digestive enzymes, gut morphology, intestinal barrier markers, gut microbiota and disease-resistance outcomes. Studies conducted only under normal rearing temperature without a thermal-challenge component were excluded unless the study objective and measured outcomes clearly addressed thermal-tolerance mechanisms.

No lower publication-year restriction was applied. Eligible reports were English-language, peer-reviewed original research articles describing controlled feeding trials, with a final publisher version of record publicly available on or before 5 July 2026. Articles assigned to a 2026 journal issue were included only when their final version of record was publicly available before the cutoff date. Publication status was verified using official publisher webpages, DOI metadata and bibliographic database records. Preprints, manuscripts under review, accepted manuscripts without a final version of record, conference abstracts, theses and dissertations, patents, institutional reports, book chapters, editorials, letters and other non-peer-reviewed or non-experimental reports were excluded. Relevant reviews were used only for background discussion and citation searching.

### 2.3. Information Sources and Search Strategy

A systematic literature search was conducted across the available coverage periods of Web of Science Core Collection, Scopus, PubMed/MEDLINE, ScienceDirect, Wiley Online Library, and SpringerLink, without applying a lower publication-year restriction. Google Scholar was used as a supplementary source to identify potentially missing records, recently published articles and citation-linked studies. The reference lists of relevant review articles and all included studies were also manually checked to identify additional eligible records. The final search was completed on 5 July 2026. Thus, 2012 was not a prespecified beginning of the search period; it was the publication year of the earliest study that met all eligibility criteria. Studies were eligible only when their final peer-reviewed version of record was publicly available on or before this date. For articles assigned to a later issue year than their initial online-publication year, eligibility was determined from the date on which the final version of record became publicly available, rather than from the year appearing in the DOI string. Publication status was verified using the official publisher webpage, DOI metadata, and bibliographic database records. The core search strategy, search fields and source-level record counts are summarized in Supplementary Table S1. However, no dedicated gray-literature database, dissertation repository, conference-proceedings source, or trial registry was searched systematically. Records of these types identified during screening were excluded in accordance with the predefined eligibility criteria. Domain experts were not contacted to identify unpublished studies or datasets.

The search strategy combined terms related to finfish, aquaculture, thermal stress, functional feed ingredients and physiological responses. Search strings were adapted to the syntax of each database. The core search strategy included terms such as “fish,” “finfish,” “cultured fish,” “farmed fish” and “aquaculture” combined with thermal-stress terms such as “thermal stress,” “heat stress,” “cold stress,” “temperature stress,” “high temperature,” “low temperature,” “temperature fluctuation” and “warming.” These terms were combined with nutrition-related terms such as “functional feed,” “functional aquafeed,” “feed additive,” “dietary supplementation,” “functional ingredient” and “nutritional strategy,” as well as outcome-related terms including “immunity,” “immune response,” “antioxidant,” “oxidative stress,” “metabolism,” “metabolic homeostasis,” “metabolomics,” “transcriptome,” “gene expression,” “cortisol,” “glucose,” “heat shock protein” and “HSP.”

Additional ingredient-specific searches were conducted to improve sensitivity. These searches included functional amino acids and related compounds, such as taurine, arginine, glutamine, methionine, tryptophan and γ-aminobutyric acid; antioxidant micronutrients, such as vitamin C, vitamin E, selenium, zinc and copper; microbial additives, such as probiotics, prebiotics, synbiotics, postbiotics, *Bacillus*, *Lactobacillus*, *Saccharomyces*, inulin, mannan oligosaccharides, fructo-oligosaccharides and β-glucan; phytogenic additives, such as plant extracts, essential oils, polyphenols, flavonoids, curcumin, thymol and carvacrol; and marine-, algal- or insect-derived bioactives, such as seaweed, algae, chitin, chitosan, nucleotides, insect meal and black soldier fly (*Hermetia illucens*). All retrieved records were exported into reference-management software, and duplicates were removed before screening. The source-specific search syntax, search fields and candidate-record counts are summarized in Supplementary Table S1. Article-level exclusion categories, the full 28-study evidence map and the risk-of-bias table are provided in Supplementary Tables S2–S4 and Supplementary File S1.

### 2.4. Study Selection

Study selection was performed in two sequential stages. First, titles and abstracts of all retrieved records were screened against the eligibility criteria. Second, full texts of potentially eligible studies were assessed to determine final inclusion. Screening was conducted independently by two reviewers. Disagreements between reviewers were resolved through discussion, and a third reviewer was consulted when consensus could not be reached. Reasons for exclusion at the full-text stage were recorded and summarized. The complete study selection process is presented using a PRISMA flow diagram, including the number of records identified, duplicates removed, records screened, full-text articles assessed for eligibility and studies included in the final synthesis.

### 2.5. Data Extraction

Data were extracted using a standardized extraction form developed before full-text assessment. The form was pilot-tested using a subset of eligible studies and refined before final extraction. Extracted information included bibliographic details, fish species, life stage, initial body weight, feeding habit, culture environment, experimental design, number of treatments, number of replicates, fish per tank, feeding duration, dietary formulation, functional-ingredient type, inclusion level, feeding rate and comparator diet. Information on the thermal-stress protocol was also extracted, including stress type, control temperature, stress temperature, exposure duration, rate of temperature change and recovery period, where available.

Thermal-stress models were classified using two complementary dimensions: thermal direction and exposure architecture. The reference temperature was defined as the concurrent control, acclimation or challenge-start temperature reported in the original study. When no concurrent reference was available, a species-specific optimal range reported in the source article was used only descriptively and was explicitly identified as a source-reported optimal range; otherwise, the reference temperature and thermal deviation were coded as not reported. Thermal deviation was calculated as ΔT = Tstress − Treference, with positive values indicating heat exposure and negative values indicating cold exposure. Exposure pattern was classified using review-specific operational criteria as rapid acute (≤24 h), short-term (>24 h to 7 days), prolonged (>7 days), endpoint-defined, or NR/unclassified. Endpoint-defined models comprised progressive warming or cooling tests in which the terminal temperature depended on a physiological, behavioral or mortality endpoint rather than a predetermined exposure duration. Multi-stressor conditions were retained as modifiers rather than treated as separate duration categories. These operational categories were developed for transparent evidence mapping and should not be interpreted as universal species-independent physiological thresholds. Study-level temperatures, ΔT values, temperature-change patterns, exposure durations, recovery phases and operational classifications are reported in Supplementary Table S3B.

Outcome data were extracted according to predefined primary domains. Growth and production outcomes included final body weight, weight gain, specific growth rate, feed conversion ratio, feed efficiency, feed intake and survival. Endocrine and cellular-stress-response outcomes included cortisol, circulating glucose measured during or after thermal challenge, lactate, heat-shock proteins and stress-response-related genes. Antioxidant and oxidative-damage outcomes included superoxide dismutase, catalase, glutathione peroxidase, glutathione, total antioxidant capacity, reactive oxygen species, malondialdehyde, lipid peroxidation and related antioxidant gene-expression markers. Immune outcomes included lysozyme activity, complement activity, immunoglobulin levels, phagocytic activity, respiratory burst, cytokines, antimicrobial peptides and immune-related genes. Metabolic and nutrient-regulatory outcomes included plasma total protein, albumin, globulin, triglycerides, cholesterol, ammonia, digestive enzymes, tissue glycogen, glucose-transport indicators such as GLUT4, lipid- and amino-acid-metabolism indicators, nutrient-metabolism-related genes and metabolomics-related endpoints. Circulating glucose measured as an acute systemic response to thermal challenge was coded within the endocrine and cellular-stress-response domain, whereas tissue-level glucose transport, glycogen regulation and glucose-metabolism pathways were coded within the metabolic and nutrient-regulatory domain. Cortisol was not coded as a metabolic outcome. IGF-1 was classified as a growth-regulatory indicator, whereas hemoglobin, hematocrit and blood-cell counts were recorded separately as hematological or systemic physiological indicators. Gut-health outcomes included intestinal morphology, goblet cells, mucin expression, tight-junction genes and gut microbiota. When studies included pathogen-challenge tests, pathogen species, challenge method, post-challenge survival and relative percent survival were also extracted.

When numerical data were presented only in figures, values were extracted using digital image-extraction software where appropriate. If essential information was missing, corresponding authors were contacted when necessary. When missing data could not be obtained, the study was retained in the qualitative evidence map if sufficient information was available for classification. Each study was also classified according to ingredient class, thermal-stress type, fish feeding habit, outcome domain and overall direction of response. For count-based evidence mapping, each measured variable was assigned to one primary outcome domain. A study could contribute to multiple domains when it reported distinct variables, but the same measured variable was not counted in more than one domain. Secondary mechanistic relationships could still be discussed narratively without changing the primary variable-level classification.

### 2.6. Risk-of-Bias and Methodological-Quality Assessment

The risk of bias of all 28 included studies was evaluated using an adapted version of SYRCLE’s risk-of-bias tool for animal intervention studies [45]. The formal assessment comprised 10 domains: sequence generation, baseline similarity, allocation concealment, random housing or tank allocation, blinding of caregivers and investigators, random outcome assessment, blinding of outcome assessors, incomplete outcome data, selective outcome reporting and other potential sources of bias. Each domain was rated as low, high or unclear risk of bias. A low-risk judgment was assigned when the published report provided sufficient information to indicate that the relevant source of bias had been adequately controlled. A high-risk judgment was assigned only when the reported design, sampling procedure or statistical analysis indicated a specific risk of bias. An unclear-risk judgment was assigned when the published report did not provide sufficient information to support either a low- or high-risk classification.

Statements that fish had been “randomly assigned” were not considered sufficient evidence of low-risk sequence generation unless the method used to generate the allocation sequence was described. Similarly, the selective-reporting domain was rated as unclear when no prospectively available study protocol or prespecified outcome record could be identified. No composite methodological-quality score was calculated because the SYRCLE domains represent distinct sources of bias and should not be treated as equally weighted components of a single numerical scale. Instead, the number and percentage of low-, unclear- and high-risk judgments were calculated separately for each domain using the 28 included studies as the denominator.

Because dietary treatments in aquaculture experiments are generally administered at tank level, the experimental unit received particular attention in the “other bias” domain. The tank was considered the appropriate experimental unit for diet-related comparisons unless the design and analysis justified another unit. A study was rated as high risk for other bias when individual fish or within-tank subsamples were treated as statistically independent replicates without accounting for tank-level treatment allocation, or when replicate tanks were combined into a single treatment-level tank during the thermal challenge. When tank allocation, subsampling or the analytical unit was insufficiently described, the judgment was unclear. Experimental-unit concerns were interpreted at the outcome level rather than automatically applied to every result within an affected study. When growth, feed-utilization or survival outcomes were calculated from independent replicate-tank means, those outcomes were retained as tank-level evidence. In contrast, biochemical, immune, histological or molecular outcomes based on multiple fish from the same tank without tank-level aggregation or an appropriate nested, hierarchical or mixed-effects model were considered affected by pseudoreplication and were interpreted as supportive or hypothesis-generating evidence. When replicate tanks or cages were combined into a single treatment-level tank during the thermal challenge, the resulting challenge-specific outcomes were regarded as unreplicated. Study-specific affected outcomes and their consequences for interpretation are provided in Supplementary Table S4C.

Three aquaculture-specific reporting domains were assessed separately from the formal SYRCLE tool: (i) tank replication and definition of the experimental unit, (ii) clarity of diet formulation and functional-ingredient dose and (iii) completeness of the thermal-stress protocol. These domains were classified as adequate, unclear or concern and were not combined with the SYRCLE judgments. Study-level SYRCLE judgments are presented in Supplementary Table S4A, study-specific explanations supporting these judgments are provided in Supplementary Table S4B, and the outcome-level consequences of experimental-unit concerns in the 14 affected studies are summarized in Supplementary Table S4C. Domain-level frequencies and percentages are presented in Supplementary Table S5, and the separate aquaculture-specific reporting assessment is provided in Supplementary Table S6. Risk-of-bias judgments informed the interpretation of the evidence but were not used as automatic exclusion criteria. An unclear judgment indicates insufficient reporting and should not be interpreted as proof that the relevant safeguard was not implemented.

### 2.7. Evidence Mapping and Data Synthesis

The primary synthesis was conducted as a systematic evidence map using a hierarchical intervention-classification framework. Interventions were first classified as single-component or combination formulations. Single-component interventions were then assigned to one mutually exclusive primary class based on material identity and source: (i) chemically defined nutrients and metabolites, (ii) microbial preparations, (iii) natural-product-derived bioactives or (iv) algal preparations. Specific subclasses included vitamins, minerals and trace elements, amino acids, lipids, organic acids, carotenoids, probiotics, botanical extracts, essential oils, bee-derived products, fungal products and microalgae. Combination formulations were classified separately and annotated according to their constituent components. Antioxidant, immunomodulatory, gut-protective and metabolic effects were coded independently as outcome or mechanistic domains rather than ingredient categories. Each study was assigned to one primary intervention class but could contribute to multiple outcome domains. Assignment to an intervention class did not itself establish functional eligibility; functional status had already been determined at the study-arm level using the operational criteria specified in Section 2.2. Studies were also categorized by fish species, feeding habit, life stage, culture environment, thermal-stress type, feeding duration and thermal-challenge protocol. The quantitative thermal-protocol fields and the corresponding operational thermal-model assignments are provided at the study level in Supplementary Table S3B. The evidence map was used to identify research clusters, knowledge gaps and under-represented species, interventions or response markers. Summary tables and figures were used to present the distribution of evidence across intervention classes and biological outcomes.

The synthesis was organized around seven major outcome domains: growth performance and survival under thermal stress; endocrine and cellular-stress response; antioxidant defense and oxidative damage; innate and mucosal immunity; metabolic and nutrient-regulatory responses; intestinal health and microbiota; and disease resistance after dietary or thermal conditioning. Each measured variable was assigned to one primary outcome domain for count-based mapping, although a study could contribute to multiple domains when it reported distinct outcomes. The same measured variable was not counted in more than one domain. The term “metabolic homeostasis” was reserved for the higher-level interpretation of coordinated maintenance or recovery across complementary metabolic indicators and was not assigned on the basis of a single biomarker. Hematological indicators were extracted separately and treated as supportive systemic responses.

To improve consistency across the outcome-domain syntheses, the findings in Section 3.5, Section 3.6, Section 3.7, Section 3.8, Section 3.9, Section 3.10 and Section 3.11 were presented using the same intervention sequence: (i) chemically defined nutrients and metabolites, (ii) microbial preparations, (iii) natural-product-derived bioactives, (iv) algal preparations and (v) combination formulations. Within each primary class, interventions were discussed by subclass and then by study, while fish species and thermal-stress model were treated as study characteristics rather than alternative organizing categories. Cross-domain effects of the same intervention were integrated in an intervention-by-outcome matrix .

For each domain, the direction of response was classified as positive, negative, neutral or mixed compared with the control group. A positive response was defined as improvement in growth, survival, feed utilization, antioxidant capacity, immune function, gut integrity or metabolic and nutrient-regulatory function. A negative response was defined as reduced performance, poorer feed utilization, increased oxidative damage, immune suppression, metabolic or nutrient-regulatory disturbance or reduced survival. A mixed response was assigned when the outcome differed by dose, tissue, sampling time, fish species or biomarker.

Because substantial heterogeneity was expected among fish species, dietary additives, feeding periods and thermal-stress models, quantitative pooling was not used as the primary synthesis approach. Where meta-analysis was not appropriate, findings were synthesized narratively according to SWiM guidance, with explicit reporting of how studies were grouped, how outcome directions were summarized and how limitations of the synthesis were handled [44].

### 2.8. Quantitative Synthesis

No formal quantitative meta-analysis was performed because the included studies differed substantially in species, functional-ingredient class, dietary inclusion level, feeding duration, thermal-stress protocol, sampled tissues and outcome reporting. The synthesis therefore prioritized structured evidence mapping, response-direction classification and mechanistic interpretation across growth, survival, stress biomarkers, antioxidant defense, immunity, gut health and metabolic indicators.

If future updates identify at least three independent studies with sufficiently comparable intervention types, control conditions and outcome metrics, exploratory random-effects meta-analysis may be added using standardized mean differences or log response ratios for continuous outcomes and risk ratios or odds ratios for survival or disease-challenge outcomes. Until such comparability is established, quantitative pooling would risk overinterpreting biologically heterogeneous evidence. Formal assessment of small-study effects or publication bias was not performed because no meta-analysis was conducted and the included studies were highly heterogeneous. Because dedicated gray-literature sources were not systematically searched and eligibility was restricted to English-language, peer-reviewed final articles, unpublished, non-English, null or negative findings may be under-represented. Accordingly, the apparent consistency of beneficial responses should be interpreted in light of the potential for language and publication bias.

### 2.9. Confidence and Translational Interpretation of Evidence

Confidence in the body of evidence was evaluated separately from the breadth of biological responses reported within individual studies. Confidence was interpreted primarily according to the number of independent studies, consistency of response direction, methodological limitations and risk of bias, validity of the experimental unit, aquaculture relevance of the species and life stage, clarity and directness of the thermal-stress model, availability of dose–response information and comparability of the reported outcomes.

Evidence was considered to have relatively higher confidence when multiple independent studies reported generally consistent effects under directly relevant thermal-stress conditions and when the principal outcomes were not dominated by serious experimental-unit concerns or other major methodological limitations. Evidence was considered moderate when findings were generally consistent but were limited by study number, species concentration, protocol heterogeneity, incomplete dose–response information or unclear risk-of-bias reporting. Evidence was considered emerging or low-confidence when it was based on one or few studies, showed mixed or inconsistent responses, involved narrow species or stress-model coverage, or depended mainly on outcomes affected by experimental-unit concerns.

The simultaneous improvement of several response domains within a study—such as growth or survival, antioxidant defense, immune regulation, gut or tissue condition and metabolic stability—was interpreted separately as cross-domain biological coherence. Such coherence may strengthen mechanistic plausibility and translational relevance, but it did not by itself increase the confidence rating of an intervention class without independent replication and adequate methodological support. Conversely, consistent evidence for an important outcome across multiple independent studies was not downgraded solely because each study measured a narrower outcome panel.

Because this review was designed as a systematic evidence map rather than a formal certainty-of-evidence assessment using GRADE or a quantitative meta-analysis, the terms higher, moderate and emerging confidence represent structured qualitative judgments rather than validated numerical scores or universal efficacy rankings.

## 3. Results

### 3.1. Study Selection

The expanded database and citation-linked search identified 386 candidate records. Of these, 361 records were retrieved from electronic database or publisher searches and 25 additional records were identified through backward and forward citation checking of relevant articles and reviews. After removal of 112 duplicates, 274 records were screened by title and abstract. A total of 203 records were excluded because they were not directly related to dietary functional interventions, finfish aquaculture, temperature-related stress models or relevant physiological outcomes. Seventy-one full-text articles were assessed for eligibility. Forty-three full-text articles were excluded for the following reasons: no thermal-stress or temperature-related challenge (*n* = 12), no dietary intervention (*n* = 8), non-finfish or non-aquaculture model (*n* = 6), review or non-original article (*n* = 7), and absence of an appropriate comparator or insufficient extractable outcomes (*n* = 10). The operational definitions and article-level decision rules for full-text exclusion are provided in Supplementary Table S2. Twenty-eight primary feeding studies met the eligibility criteria and were retained in the qualitative evidence map. No lower publication-year limit had been applied, and the eligible studies had publication years ranging from 2012 to 2026. The two articles assigned to a 2026 bibliographic year had both been published as final peer-reviewed versions of record before the search cutoff of 5 July 2026. No preprints, manuscripts under review, or unpublished accepted manuscripts were included. Because the eligible studies differed substantially in species, stress model, functional-ingredient class, feeding duration and outcome reporting, selected representative mechanistic studies with direct thermal-stress dietary interventions and extractable cross-domain outcomes were highlighted in the main synthesis tables, while the full evidence base was used to support the evidence-gap and translational synthesis. Therefore, the results should be interpreted as a qualitative evidence map and mechanistic synthesis rather than a quantitative estimate of additive-specific effect size.

### 3.2. General Characteristics of Included Studies

The publication years of the 28 eligible studies ranged from 2012 to 2026, reflecting increasing interest in feed-based strategies for improving thermal-stress resilience in cultured finfish. This was the observed range of eligible publications rather than a prespecified search window. The evidence base included studies conducted under heat stress, cold stress, sub-optimal temperature, elevated-temperature challenge and multi-stressor temperature models. Nile tilapia, *Oreochromis niloticus*, was the most frequently represented species, especially in studies evaluating sodium butyrate, organic selenium, dietary lipid strategies, curcumin and nano-curcumin, propolis, alpha-lipoic acid, *Chlorella vulgaris*, vitamin E, *Pelargonium sidoides*, bay laurel essential oil, white button mushroom and *Spirulina*-based supplementation under thermal-stress conditions [36,46,47,48,49,50,51,52,53,54,55,56,57]. Other cultured finfish species included striped catfish, *Pangasianodon hypophthalmus*; rohu, *Labeo rohita*; rainbow trout, *Oncorhynchus mykiss*; Asian seabass, *Lates calcarifer*; European seabass, *Dicentrarchus labrax*; pufferfish, *Takifugu obscurus*; Wuchang bream, *Megalobrama amblycephala*; largemouth bass, *Micropterus salmoides*; and hybrid crucian carp HCC2. These species were used to evaluate selenium nanoparticles, riboflavin, dietary copper, probiotic mixtures, grape extract, hyssop extract, *Abies sibirica* essential oil, astaxanthin, taurine, emodin with vitamin C, vitamin C, tea polyphenols and dietary *Spirulina* supplementation [58,59,60,61,62,63,64,65,66,67,68,69,70,71].

Most studies used juvenile fish, whereas other production stages were less frequently represented. Among studies reporting the relevant variables, total allocation ranged from 120 to 1500 fish (27/28 studies), with two to six independent tanks or cages per treatment (26/28) and 8–100 fish per experimental unit (26/28); three replicate units per treatment were most common (22 studies). Feeding periods ranged from 9 to 105 days (28/28). Reported experimental diets contained 27.43–55.3% crude protein (25/28) and 3.0–18.92% crude lipid (24/28). Common protein sources included fish meal, soybean meal and corn gluten or meal, whereas fish and plant oils were the principal lipid sources. Variables not reported in the source articles are identified as “NR” in Supplementary Table S3A of Supplementary File S1. Supplementary Table S3 provides the representative outcome-level extraction, while Supplementary Table S3A and the complete evidence map in Supplementary File S1 provide the study-level design and diet characteristics for all 28 studies. The class-level synthesis of the evidence is presented in Table 1.

### 3.3. Evidence Map of Functional-Aquafeed Strategies

**Figure 2:**
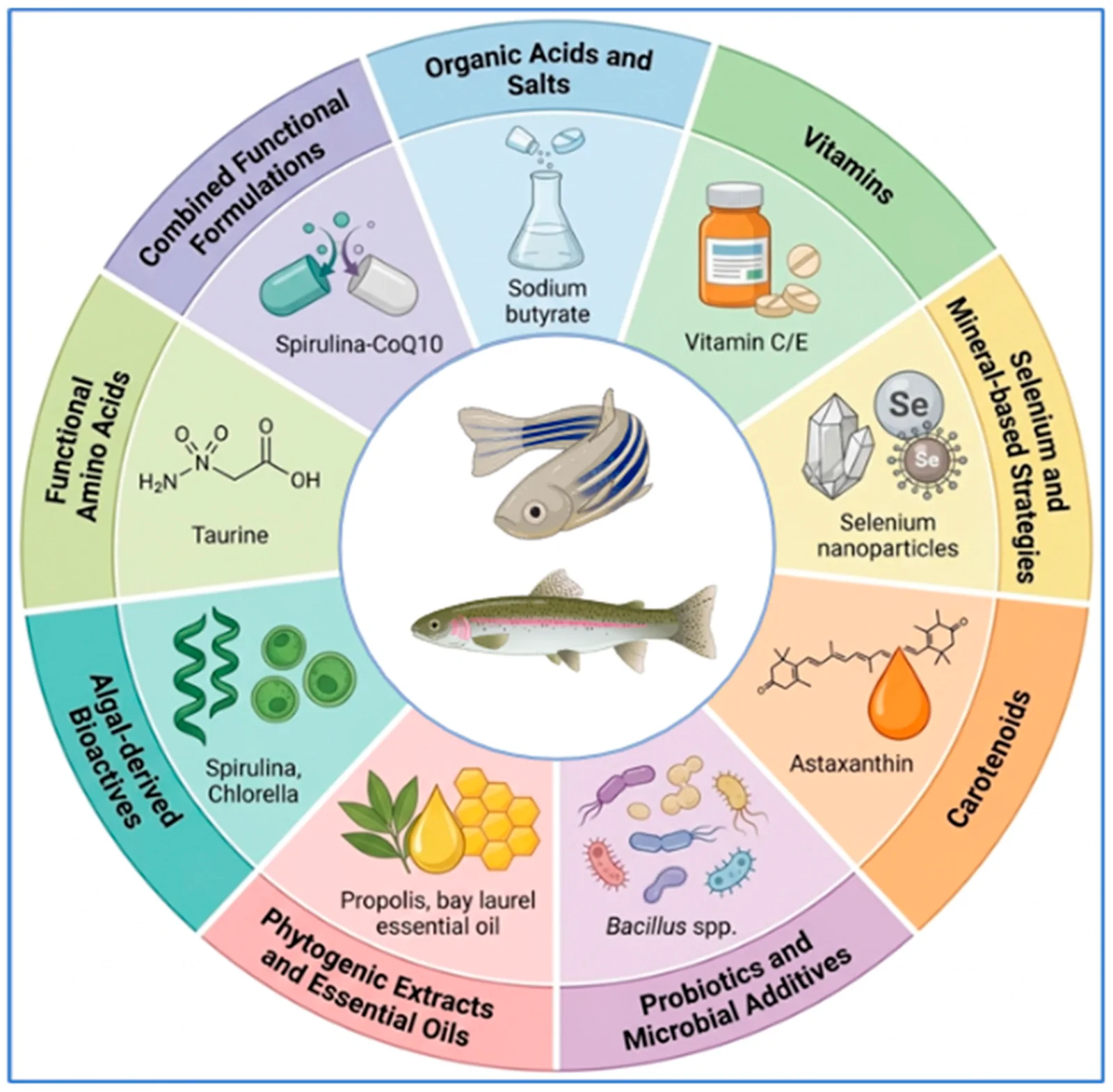
The hierarchical classification of functional-aquafeed interventions evaluated for thermal-stress resilience in cultured finfish. At the first level, interventions were classified as single-component or combination formulations. Single-component interventions were subsequently assigned to chemically defined nutrients and metabolites, microbial preparations, natural-product-derived bioactives or algal preparations. Specific subclasses included vitamins, minerals and trace elements, amino acids, lipids, organic acids, carotenoids, probiotics, botanical extracts, essential oils, bee-derived products, fungal products and microalgae. Combination formulations were classified separately and annotated according to their constituent components. Antioxidant, immune, gut-protective and metabolic functions were evaluated as overlapping mechanistic outcome domains rather than as ingredient classes.

Using the predefined hierarchical framework, the included interventions were assigned to five mutually exclusive primary classes: chemically defined nutrients and metabolites, microbial preparations, natural-product-derived bioactives, algal preparations and combination formulations. Chemically defined nutrients and metabolites included vitamins, minerals and trace elements, amino acids, lipids, organic acids and carotenoids. Microbial preparations included single- or multi-strain probiotic interventions. Natural-product-derived bioactives included botanical extracts, essential oils, propolis and fungal-derived products, whereas algal preparations included single-source *Chlorella* or *Spirulina* interventions. Formulations containing two or more distinct active components were classified separately as combination formulations. Functional properties such as antioxidant, immunomodulatory, gut-protective or metabolic activity were treated as mechanistic outcome domains rather than ingredient categories. This framework prevented duplicate classification. For example, organic selenium and nano-selenium administered alone were classified within the mineral and trace-element subclass of chemically defined nutrients and metabolites, whereas selenium nanoparticles combined with riboflavin were classified as a mineral–vitamin combination formulation. The major intervention classes identified in the evidence map are summarized schematically in Figure 2.

Vitamin- and mineral-based interventions included vitamin C, vitamin E, organic selenium, selenium nanoparticles, riboflavin and dietary copper, which were evaluated for their roles in growth, immune response, antioxidant defense, stress biomarkers and heat-shock protein regulation under sub-optimal, high-temperature or multi-stressor conditions [47,54,58,62,63,68,69]. These nutrients were not considered functional merely because they were essential nutrients or normal dietary components. They were included only when their dietary levels were deliberately manipulated as experimental treatments and their effects were evaluated specifically in relation to thermal-stress resilience. Accordingly, the vitamin C studies [58,69] were retained because vitamin C supplementation was experimentally tested against an appropriate comparator for high-temperature or heat-stress-related responses rather than being present only as a routine component of the basal diet. Carotenoid and functional amino acid supplementation, particularly astaxanthin and taurine, were evaluated for high- or low-temperature stress mitigation through antioxidant defense, non-specific immunity, calcium homeostasis, apoptosis regulation and stress-related molecular responses [60,61].

Microbial-based strategies included dietary *Bacillus* spp. and a dietary probiotic mixture in rohu. These studies mainly evaluated protection against heat shock or elevated-temperature exposure through immune regulation, antioxidant responses, histopathological protection, apoptosis-related responses and heat-shock protein expression [59,72]. Phytogenic strategies included propolis, hyssop extract, grape extract, curcumin and nano-curcumin, *P. sidoides* extract, bay laurel essential oil and *A. sibirica* essential oil, which were investigated under cold-stress, heat-stress or broader thermal-stress conditions for their effects on growth, antioxidant defense, immune response, metabolic indicators and biochemical stress markers [50,51,55,56,64,65,66]. Organic acid-based intervention was represented by sodium butyrate supplementation, which was evaluated in Nile tilapia exposed to heat stress and was linked with growth, intestinal morphology, immune response, antioxidant status, cortisol, glucose and HSP70 regulation [36]. Single-source algal preparations included *Chlorella vulgaris* and dietary *Spirulina platensis*, which were evaluated under cold/hypoxia and heat-stress conditions, respectively [53,71]. In Mahfuj et al. [71], the probiotic was administered through the rearing water in a separate treatment arm and was therefore excluded from the dietary intervention classification. The *Spirulina*–coenzyme Q10 nanoemulsion was classified separately as a combination formulation [57].

Using the predefined hierarchical classification framework, all 28 included studies were assigned to mutually exclusive primary intervention classes. Overall, the evidence map indicated that antioxidant, immune and growth-related outcomes were the most frequently evaluated domains. In contrast, integrated multiomics approaches, long-term recovery responses, repeated temperature-fluctuation models and combined thermal-stress–disease-challenge designs were less consistently represented. Therefore, evidence confidence differed among intervention classes and should be interpreted according to species coverage, thermal-protocol clarity, dose–response design, outcome consistency and risk-of-bias reporting. Table 1 summarizes the number of contributing studies, representative interventions, thermal-stress contexts, major outcome domains, overall response patterns and principal limitations for each intervention class.

**Figure 3:**
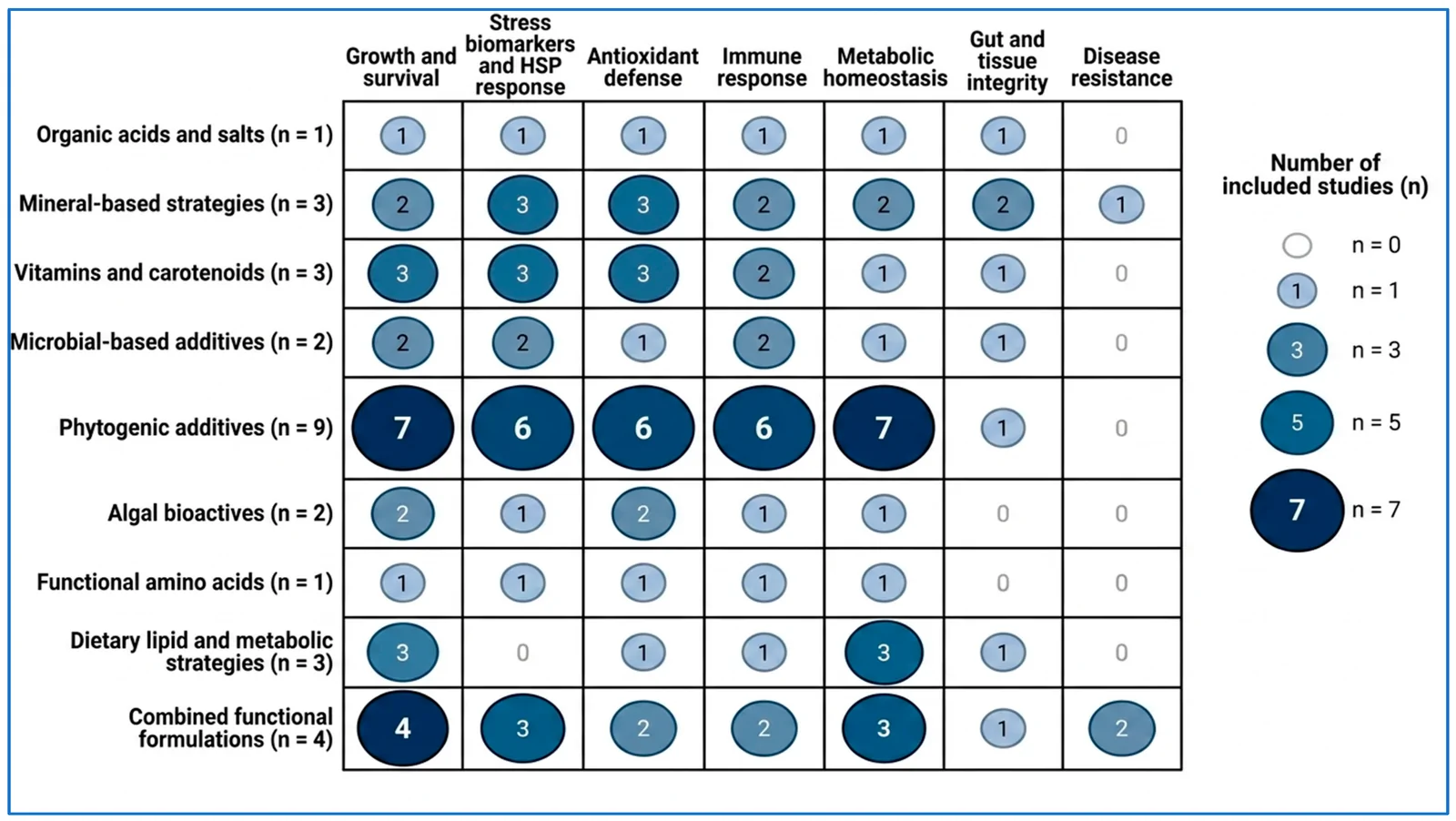
A count-based evidence map linking mutually exclusive intervention subclasses or grouped subclasses with thermal-stress-response domains. The rows represent intervention subclasses or grouped subclasses assigned according to the hierarchical classification framework, whereas the columns represent the seven predefined outcome domains. Each study was assigned to one mutually exclusive primary intervention class but could contribute to multiple outcome domains. Combination formulations were treated as a separate primary class and annotated according to their constituent components.

Beyond the tabular synthesis, Figure 3 presents an ingredient-by-outcome evidence-map matrix that illustrates the distribution of functional-aquafeed categories across major physiological response domains. This matrix enables rapid comparison of relative evidence coverage for growth and survival, endocrine and cellular-stress responses, antioxidant defense, immune regulation, metabolic and nutrient-regulatory responses, gut health and disease resistance.

### 3.4. Thermal-Stress Models Used in Included Studies

Application of the predefined operational criteria showed that seven of the 28 studies used rapid acute challenges lasting ≤24 h, six used short-term exposures lasting >24 h to 7 days, 11 used prolonged exposures lasting >7 days, and three used endpoint-defined thermal-tolerance tests. One study could not be assigned to a duration class because the reference condition and exposure duration were not sufficiently reported. Thermal protocols included direct temperature transfer, progressive warming or cooling, ramp-and-hold exposure, sustained non-optimal-temperature rearing and critical or lethal endpoint tests. Thermal deviations ranged from relatively moderate fixed differences, such as −7 to −8 °C or +6 to +9 °C, to larger acute deviations and progressive challenges that continued until critical or lethal endpoints. Several studies also combined temperature with arsenic exposure, low pH, hypoxia or salinity stress, or applied a pathogen challenge after thermal conditioning. Complete study-level protocol data, including reference temperature, stress temperature, ΔT, temperature-change pattern, exposure duration, recovery phase and operational classification, are presented in Supplementary Table S3B.

**Figure 4:**
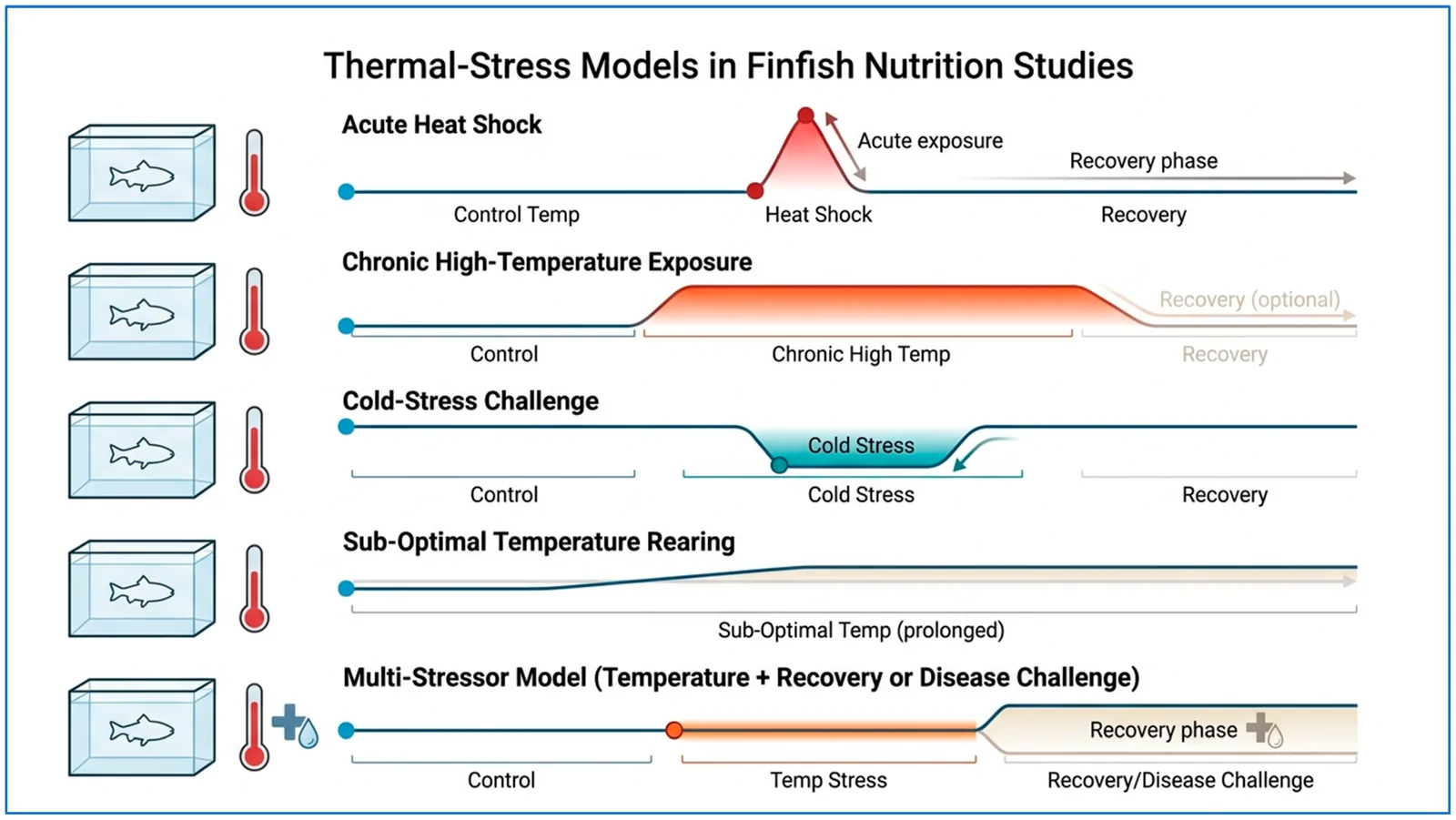
The operational classification of the thermal-stress models used in the 28 included functional-aquafeed studies. The models were classified according to exposure duration and protocol architecture as rapid acute (≤24 h), short-term (>24 h to 7 days), prolonged (>7 days), endpoint-defined, or NR/unclassified. Endpoint-defined models comprised progressive warming or cooling tests in which the terminal temperature depended on a physiological, behavioral or mortality endpoint. The models were further characterized according to heat, cold or bidirectional exposure; fixed, direct-transfer, gradual or ramp-and-hold temperature change; and the presence of additional stressors or post-thermal challenges. These categories are review-specific evidence-mapping definitions rather than universal species-independent physiological thresholds. Quantitative study-level details are provided in Supplementary Table S3B.

Thermal-stress models varied considerably across the included studies. Heat-stress models included acute heat exposure, chronic elevated-temperature rearing and post-feeding thermal challenge. For example, sodium butyrate and white button mushroom were evaluated in Nile tilapia subjected to heat-stress resistance tests after feeding periods, while selenium nanoparticles with riboflavin and dietary copper were evaluated in striped catfish exposed to elevated-temperature multi-stressor models [36,46,62,63]. Astaxanthin, emodin with vitamin C, vitamin C, tea polyphenols, *P. sidoides* extract and *Spirulina*-coenzyme Q10 nanoemulsion were also examined in relation to high-temperature or heat-stress responses in finfish [55,57,58,60,69,70]. Recent studies further evaluated dietary *Bacillus* spp., probiotics, *Spirulina*-based supplements and essential-oil products under heat-shock or thermal-stress conditions [59,65,71,72]. Because the included studies differed substantially in control temperature, challenge temperature, exposure duration, temperature-shift rate and recovery assessment, the main thermal-stress models were classified schematically in Figure 4.

Cold-stress and sub-optimal-temperature models were also represented. Organic selenium was evaluated in Nile tilapia reared under sub-optimal temperature, while dietary lipid levels, dietary lipid sources, taurine, propolis, alpha-lipoic acid, *C. vulgaris*, grape extract and bay laurel essential oil were tested under cold-stress, low-temperature or cold-tolerance challenge conditions [47,48,49,51,52,53,56,61,66]. Compared with heat-stress studies, cold-stress studies were fewer and were mainly concentrated in Nile tilapia, although Asian seabass and pufferfish provided additional species-level evidence. Temperature-fluctuation and recovery models were less common than constant heat or cold exposure. Differences in control temperature, stress temperature, exposure duration, rate of temperature change and recovery period created substantial heterogeneity among studies. The thermal-stress-model patterns and their implications for interpretation are synthesized in Table 2.

To facilitate comparison across outcome domains, Section 3.5, Section 3.6, Section 3.7, Section 3.8, Section 3.9, Section 3.10 and Section 3.11 retain a consistent intervention sequence—chemically defined nutrients and metabolites, microbial preparations, natural-product-derived bioactives, algal preparations and combination formulations—while fish species and thermal conditions are described within the relevant study narratives.

### 3.5. Effects on Growth Performance, Feed Utilization and Survival

Growth performance and feed utilization were among the most frequently reported outcomes, whereas survival was measured less consistently. Among chemically defined nutrients and metabolites, sodium butyrate improved growth performance and feed efficiency in Nile tilapia before heat exposure and was associated with higher survival after the challenge [36]. Organic selenium improved growth-related responses under sub-optimal temperature [47], while dietary lipid source and level produced source- or dose-dependent effects on growth, liver condition and cold tolerance [48,49]. Alpha-lipoic acid, vitamin E, astaxanthin, taurine and vitamin C were also linked with improved growth, feed utilization, physiological protection or thermal tolerance in the relevant species and stress models [52,54,60,61,69].

Evidence from microbial preparations was limited to two studies: a dietary probiotic mixture improved growth-related performance in rohu reared at higher temperature, and dietary *Bacillus* spp. produced protective growth- and health-related responses in heat-shocked Nile tilapia [59,72]. Natural-product-derived bioactives, including white button mushroom, curcumin or nano-curcumin, propolis, *P. sidoides*, bay laurel essential oil, hyssop extract, *A. sibirica* essential oil, grape extract and tea polyphenols, showed beneficial or dose-dependent effects on growth, feed utilization, heat- or cold-stress resistance, or survival-related outcomes [46,50,51,55,56,64,65,66,70]. Among algal preparations, *C. vulgaris* or its nanoparticle preparation supported performance during cold and hypoxia exposure, whereas dietary *S. platensis* improved growth-related responses in rohu maintained under thermal stress [53,71].

Combination formulations also produced growth- and survival-related responses. Selenium nanoparticles with riboflavin improved growth, feed efficiency, protein efficiency ratio and specific growth rate in striped catfish exposed to elevated temperature and arsenic [62], while *Spirulina*–coenzyme Q10 nanoemulsion improved growth and survivability in heat-stressed Nile tilapia [57]. Emodin with vitamin C and the multicomponent formulation used in European seabass contributed mainly mixed or mechanistic performance evidence [58,67]. Across intervention classes, growth and feed-utilization benefits were most biologically coherent when the same dietary treatment also supported antioxidant defense, immune regulation, intestinal or tissue condition, or moderation of stress responses. Survival evidence was concentrated in a smaller number of studies, particularly those evaluating sodium butyrate and the two combination formulations with subsequent challenge outcomes [36,57,62]. Cross-domain breadth was interpreted as biological coherence rather than as an independent increase in evidence confidence.

### 3.6. Endocrine and Cellular-Stress Responses

Endocrine and cellular-stress outcomes included cortisol, circulating glucose, lactate, heat-shock proteins and related stress-response genes. Circulating glucose measured during or after thermal challenge was retained in this domain because it reflected systemic stress-related energy mobilization. Among chemically defined nutrients and metabolites, sodium butyrate reduced circulating glucose and cortisol and altered hepatic HSP70 expression in heat-stressed Nile tilapia [36]. Organic selenium modulated stress-related responses under sub-optimal temperature [47], whereas taurine influenced stress-related molecular pathways during low-temperature exposure [61].

Microbial preparations were represented by dietary *Bacillus* spp., which altered heat-shock protein gene expression and other stress indicators during acute heat shock in Nile tilapia, and by a probiotic mixture that influenced HSP70 and apoptosis-related responses in rohu at higher temperature [59,72]. Natural-product-derived bioactives, including *P. sidoides*, hyssop extract, *A. sibirica* essential oil, grape extract and tea polyphenols, were evaluated for physiological or molecular responses to heat or cold stress, although direct endocrine outcomes were less consistently reported than antioxidant, immune or metabolic responses [55,64,65,66,70]. The single-source *Chlorella* and *Spirulina* studies focused mainly on performance, antioxidant, hepatic and immune responses, and therefore provided limited direct endocrine or cellular-stress evidence [53,71].

Among the combination formulations, selenium nanoparticles with riboflavin reduced circulating glucose, serum cortisol and HSP70 expression in striped catfish under elevated temperature and arsenic exposure [62]. Emodin with vitamin C modified HSP70 mRNA expression in Wuchang bream [58], while the multicomponent supplement used in European seabass altered HSP70 and other stress-related responses during heatwave exposure [67]. Heat-shock protein findings were interpreted together with cortisol, circulating glucose, antioxidant status, tissue condition and survival because increased HSP expression may indicate either enhanced cellular protection or greater cellular stress. HSP outcomes were therefore not interpreted as protective in isolation.

### 3.7. Antioxidant Defense and Oxidative Damage

Antioxidant defense was one of the most consistently evaluated domains. Among chemically defined nutrients and metabolites, sodium butyrate increased superoxide dismutase, catalase and glutathione peroxidase activities and reduced malondialdehyde before and after heat stress [36]. Organic selenium improved antioxidative capacity under sub-optimal temperature [47], whereas dietary nano-selenium, copper, vitamin E, astaxanthin, taurine and alpha-lipoic acid supported antioxidant defense or reduced oxidative damage under heat, cold or multi-stressor conditions [52,54,60,61,63,68].

Microbial preparations also contributed antioxidant evidence: *Bacillus* spp. improved antioxidant and histopathological responses during heat shock, while the probiotic mixture in rohu was associated with antioxidant, hemato-immunological and apoptosis-related responses at higher temperature [59,72]. Natural-product-derived bioactives including white button mushroom, curcumin or nano-curcumin, propolis, *P. sidoides*, bay laurel essential oil, hyssop extract, *A. sibirica* essential oil, grape extract and tea polyphenols generally improved redox status, antioxidant enzymes or resistance to oxidative damage, although effects varied with dose, source and model [46,50,51,55,56,64,65,66,70]. *C. vulgaris* and dietary *S. platensis* enhanced antioxidant responses under cold/hypoxia or heat-stress conditions, respectively [53,71].

Combination formulations showed similarly protective redox patterns. Selenium nanoparticles with riboflavin enhanced antioxidant status and reduced lipid peroxidation in multiple tissues of striped catfish [62], and the *Spirulina*–coenzyme Q10 nanoemulsion improved antioxidant biomarkers in heat-stressed Nile tilapia [57]. Emodin with vitamin C and the European seabass multicomponent formulation contributed more context-dependent biochemical or molecular evidence [58,67]. Across intervention classes, the strongest protective interpretation occurred when increased SOD, CAT, GPx, GSH or total antioxidant capacity was accompanied by lower ROS, malondialdehyde or lipid peroxidation. Antioxidant enzyme induction alone was not considered sufficient evidence of reduced oxidative injury.

### 3.8. Innate, Mucosal and Inflammatory Immune Responses

Immune outcomes included lysozyme, complement, immunoglobulins, phagocytosis, respiratory burst, myeloperoxidase, nitroblue tetrazolium activity, cytokines, antimicrobial genes and mucosal indicators. Among chemically defined nutrients and metabolites, sodium butyrate increased phagocytic index, phagocytic activity and lysozyme activity before and after heat stress [36]. Organic selenium improved immune responses under sub-optimal temperature [47], whereas vitamins, copper, astaxanthin, taurine and other chemically defined interventions provided additional context-dependent immune or inflammatory evidence [54,60,61,63,69].

Microbial preparations were strongly associated with immune regulation. *Bacillus* spp. affected immune, antioxidant, histopathological and HSP-related responses during heat shock in Nile tilapia, whereas the probiotic mixture improved hemato-immunological status in rohu reared at higher temperature [59,72]. Natural-product-derived bioactives, including white button mushroom, curcumin or nano-curcumin, propolis, *P. sidoides*, bay laurel essential oil, hyssop extract, *A. sibirica* essential oil and related plant products, generally enhanced innate immune markers or reduced excessive inflammatory responses depending on dose and sampling phase [46,50,51,55,56,64,65]. *C. vulgaris* supported systemic condition during cold/hypoxia exposure, and dietary *S. platensis* enhanced immunity in rohu under thermal stress [53,71].

Among combination formulations, selenium nanoparticles with riboflavin increased nitroblue tetrazolium activity, total immunoglobulin, myeloperoxidase and globulin in striped catfish [62]. The *Spirulina*–coenzyme Q10 nanoemulsion improved immune biomarkers in heat-stressed Nile tilapia, while the other combination formulations contributed more limited or mechanistic immune evidence [57,58,67]. Across intervention classes, the desirable pattern was balanced immune regulation rather than universal immune stimulation. Increased basal defense markers and reduced stress-induced inflammatory responses were interpreted as potentially complementary outcomes according to tissue, sampling time and stress severity.

### 3.9. Metabolic and Nutrient-Regulatory Responses

Metabolic and nutrient-regulatory outcomes included circulating protein and lipid indices, nitrogen-metabolism indicators, digestive and absorptive measures, nutrient-transport markers, metabolism-related genes and metabolomic endpoints. Cortisol and circulating glucose measured during or after thermal challenge were synthesized in Section 3.6 and were not counted again in this domain. Among chemically defined nutrients and metabolites, sodium butyrate improved blood total protein, while its cortisol and circulating-glucose effects were retained as endocrine stress outcomes [36]. Organic selenium improved serum biochemical indices under sub-optimal temperature [47], and dietary lipid source and level altered growth, liver condition and cold tolerance, supporting a role for energy supply and membrane lipid composition in low-temperature adaptation [48,49].

The microbial studies primarily emphasized hemato-immunological, antioxidant, HSP, apoptosis and tissue outcomes, and directly comparable metabolic and nutrient-regulatory endpoints were limited [59,72]. Among natural-product-derived bioactives, bay laurel essential oil increased GLUT4 expression during cold stress, whereas IGF-1 was treated as a growth-regulatory indicator [56]. *A. sibirica* essential oil was evaluated for digestive-enzyme responses during heat stress, and tea polyphenols modified hepatic lipid-metabolism responses during acute heat stress [65,70]. *C. vulgaris* was evaluated for hepatic-function and performance-related responses during cold/hypoxia exposure, while dietary *Spirulina* mainly contributed growth, antioxidant and immune evidence under heat stress [53,71].

Combination formulations provided additional metabolic evidence. Selenium nanoparticles with riboflavin increased vitamin C concentrations in brain and muscle, while cortisol and circulating glucose were retained as stress-response outcomes [62]. Emodin with vitamin C altered biochemical parameters; the European seabass formulation was evaluated through metabolic, cellular and molecular responses; and *Spirulina*–coenzyme Q10 provided supportive biochemical and tissue evidence [57,58,67]. Overall, a single metabolite, biochemical index or metabolism-related gene was not considered sufficient evidence of metabolic homeostasis. Integrated interpretation required complementary evidence of nutrient transport, digestion, biochemical status, energy-metabolism pathways or recovery toward the unstressed condition. The outcome-centered mechanistic synthesis is provided in Table 3.

### 3.10. Gut Health, Intestinal Barrier Function and Microbiota

Gut, barrier and tissue outcomes were reported in fewer studies than growth, antioxidant or immune responses. Among chemically defined nutrients and metabolites, sodium butyrate improved intestinal histomorphology, including villus-related measurements and goblet-cell abundance, and these changes accompanied improved immune and heat-stress responses in Nile tilapia [36]. Dietary nano-selenium alleviated heat-stress-induced intestinal damage and altered intestinal antioxidant capacity and microbiota in rainbow trout [68].

Microbial preparations were represented by *Bacillus* spp., which improved histopathological status during heat shock, and by a probiotic mixture that influenced hemato-immunological and apoptosis-related responses in rohu at higher temperature [59,72]. Direct integration of microbiota, barrier markers and nutrient absorption was nevertheless limited. Natural-product-derived bioactive studies more commonly reported systemic antioxidant, immune or growth outcomes than direct intestinal barrier or microbiota endpoints; consequently, gut-mediated effects for this class were treated as plausible but incompletely resolved rather than inferred from systemic responses alone. The single-source *Chlorella* and *Spirulina* studies also provided performance, hepatic, antioxidant or immune evidence, but extractable microbiota and tight-junction outcomes were not consistently available [53,71].

Among combination formulations, *Spirulina*–coenzyme Q10 nanoemulsion improved histopathological conditions in the liver, intestine and spleen of heat-stressed Nile tilapia [57], whereas the other combination formulations provided limited direct gut or microbiota evidence. These findings support the intestine as a potential interface linking feed additives with nutrient utilization, mucosal immunity and systemic thermal resilience. However, further studies should integrate intestinal morphology, mucin and tight-junction markers, digestive function, mucosal immunity, microbiota and metabolite profiles within the same replicated design.

### 3.11. Disease Resistance After Thermal-Stress or Dietary Conditioning

Disease resistance was the least represented outcome domain. Among chemically defined nutrients and metabolites, dietary copper improved relative percentage survival and reduced cumulative mortality after *Aeromonas hydrophila* challenge in striped catfish previously reared under high-temperature multi-stressor conditions [63]. Directly relevant evidence was also provided by two combination formulations, whereas comparable post-thermal pathogen-challenge outcomes were not available for microbial preparations, natural-product-derived bioactives or algal preparations under the predefined disease-resistance criteria.

Selenium nanoparticles with riboflavin improved relative survival and reduced cumulative mortality after bacterial challenge in striped catfish previously exposed to elevated temperature and arsenic [62]. *Spirulina*–coenzyme Q10 nanoemulsion improved post-challenge survivability against *Streptococcus agalactiae* in heat-stressed Nile tilapia, particularly at higher dietary inclusion levels [57]. Together with the dietary copper study [63], these findings suggest that selected single-component and multicomponent strategies may support post-stress disease resistance when antioxidant, immune and tissue-protective responses improve concurrently. Nevertheless, conclusions remain preliminary because only three studies provided directly relevant challenge evidence, the interventions and stress models differed substantially, and experimental-unit concerns affected some challenge outcomes.

To facilitate intervention-centered interpretation across Section 3.5, Section 3.6, Section 3.7, Section 3.8, Section 3.9, Section 3.10 and Section 3.11, Table 4 presents a cross-domain matrix showing which growth, endocrine or cellular stress, antioxidant, immune, metabolic, gut or tissue, and disease-resistance outcomes were reported for representative interventions. This matrix complements Table 3 by allowing the biological response profile of the same intervention to be followed horizontally across domains.

### 3.12. Risk of Bias and Methodological Quality

All 28 included studies were assessed across 10 SYRCLE domains, yielding 280 domain-level judgments. Of these, 71 (25.4%) were rated as low risk, 195 (69.6%) as unclear risk and 14 (5.0%) as high risk. The predominance of unclear judgments mainly reflected incomplete methodological reporting rather than demonstrated methodological failure.

Baseline similarity was rated as low risk in all 28 studies (100.0%), because the experimental groups were reported to contain fish of comparable initial size and to be maintained under comparable baseline conditions. In contrast, sequence generation, allocation concealment, caregiver or investigator blinding, outcome-assessor blinding and selective outcome reporting were rated as unclear in all 28 studies (100.0%). Although many articles stated that fish were randomly allocated, none described the actual sequence-generation procedure in sufficient detail, and allocation concealment and blinding were generally not reported. Random housing or tank allocation was rated as low risk in 15 studies (53.6%) and unclear in 13 (46.4%). Random outcome assessment was low risk in six studies (21.4%) and unclear in 22 (78.6%). Incomplete outcome data were judged low risk in 20 studies (71.4%) and unclear in eight (28.6%); no study received a high-risk judgment in this domain.

The only high-risk judgments occurred in the “other bias” domain, which was rated as low risk in two studies (7.1%), unclear in 12 (42.9%) and high risk in 14 (50.0%). The experimental-unit concerns and their interpretive consequences were evaluated separately for each affected study. In 13 studies, dietary treatments were administered at tank level, but fish-level biochemical, immune, histological or molecular observations were analyzed without adequately accounting for tank-level clustering. In the remaining study, independent cage replication was lost when the fish were combined into one treatment-level tank during the thermal challenge. These concerns did not necessarily invalidate independently analyzed tank-level growth or feed-utilization outcomes from the same studies; however, affected mechanistic outcomes were interpreted cautiously and were not treated as equivalent to evidence based on independent tank replicates. The specific concern, affected outcomes and resulting interpretation for each of the 14 studies are reported in Supplementary Table S4C.

The separate aquaculture-specific assessment produced a similar pattern. Tank replication and experimental-unit reporting were considered adequate in two studies (7.1%), unclear in 12 (42.9%) and a methodological concern in 14 (50.0%). By comparison, diet formulation and dose reporting and thermal-protocol reporting were each adequate in 26 studies (92.9%) and unclear in two studies (7.1%). Thus, ingredient and temperature descriptions were generally more complete than the reporting of allocation, blinding and the analytical treatment of tank subsamples. Complete study-level SYRCLE judgments are provided in Supplementary Table S4A, supporting study-specific explanations are provided in Supplementary Table S4B, quantitative domain summaries are presented in Supplementary Table S5, and aquaculture-specific reporting judgments are presented in Supplementary Table S6A,B.

### 3.13. Evidence Gaps and Research Clusters

The evidence map revealed several clear research clusters. Most PubMed-indexed studies were concentrated around Nile tilapia and focused on heat stress, cold stress or sub-optimal temperature using functional ingredients such as sodium butyrate, selenium, probiotics, phytogenic additives and algal-derived bioactives [36,47,51,56,57,72]. Antioxidant defense, innate immunity, growth performance and stress biomarkers were the most frequently evaluated outcome domains. In contrast, temperature fluctuation, long-term recovery, broodstock nutrition, metabolomics, transcriptomics, microbiome analysis and combined thermal-stress–disease-challenge models were less represented.

Several methodological gaps were identified. First, thermal-stress protocols were not standardized across studies, limiting direct comparison among dietary interventions. Second, many studies measured only selected biomarkers and did not integrate growth, antioxidant defense, immunity, metabolism, gut health and disease resistance within the same experimental design, which limited assessment of cross-domain biological coherence but did not, by itself, determine confidence in the overall evidence. Third, dose–response studies were available for some additives, such as sodium butyrate, organic selenium, selenium nanoparticles, propolis and essential oils, but not consistently across all functional-ingredient classes [36,47,51,56,62]. Fourth, most studies focused on short- or medium-term responses, whereas long-term validation under commercial farming conditions was limited. The main evidence gaps and methodological priorities are summarized in Table 5.

Overall, the evidence suggests that selected functional aquafeeds have potential to support climate-resilient finfish culture by modulating antioxidant defense, immune function, metabolic stability, gut health and survival under thermal-stress conditions. Several interventions showed biologically coherent multisystem response patterns, including reduced cortisol or glucose, enhanced antioxidant enzyme activity, improved immune markers, reduced oxidative damage, improved intestinal morphology and higher survival after heat or disease challenge [36,57,62,72]. These coordinated responses were interpreted as cross-domain biological and translational coherence rather than as an independent measure of evidence confidence. Confidence in each intervention class was judged primarily according to the number of independent studies, consistency of response direction, methodological quality and risk of bias, experimental-unit validity, species and thermal-model relevance, thermal-protocol clarity and dose–response support. Therefore, an intervention supported by a single multisystem study remained emerging evidence, whereas greater confidence required consistent support from multiple independent studies, even when individual studies evaluated narrower outcome panels. Accordingly, confidence in the available evidence differed among intervention classes, fish species and thermal-stress models. These findings should therefore be interpreted as evidence-mapping support for promising strategies rather than universal proof of efficacy across all cultured finfish. Future studies should use standardized thermal-challenge protocols, integrated biomarker panels, dose–response designs, multiomics approaches and post-stress recovery assessments to support practical formulation of climate-resilient aquafeeds for cultured finfish.

## 4. Discussion

### 4.1. Overview of the Evidence Landscape

**Figure 5:**
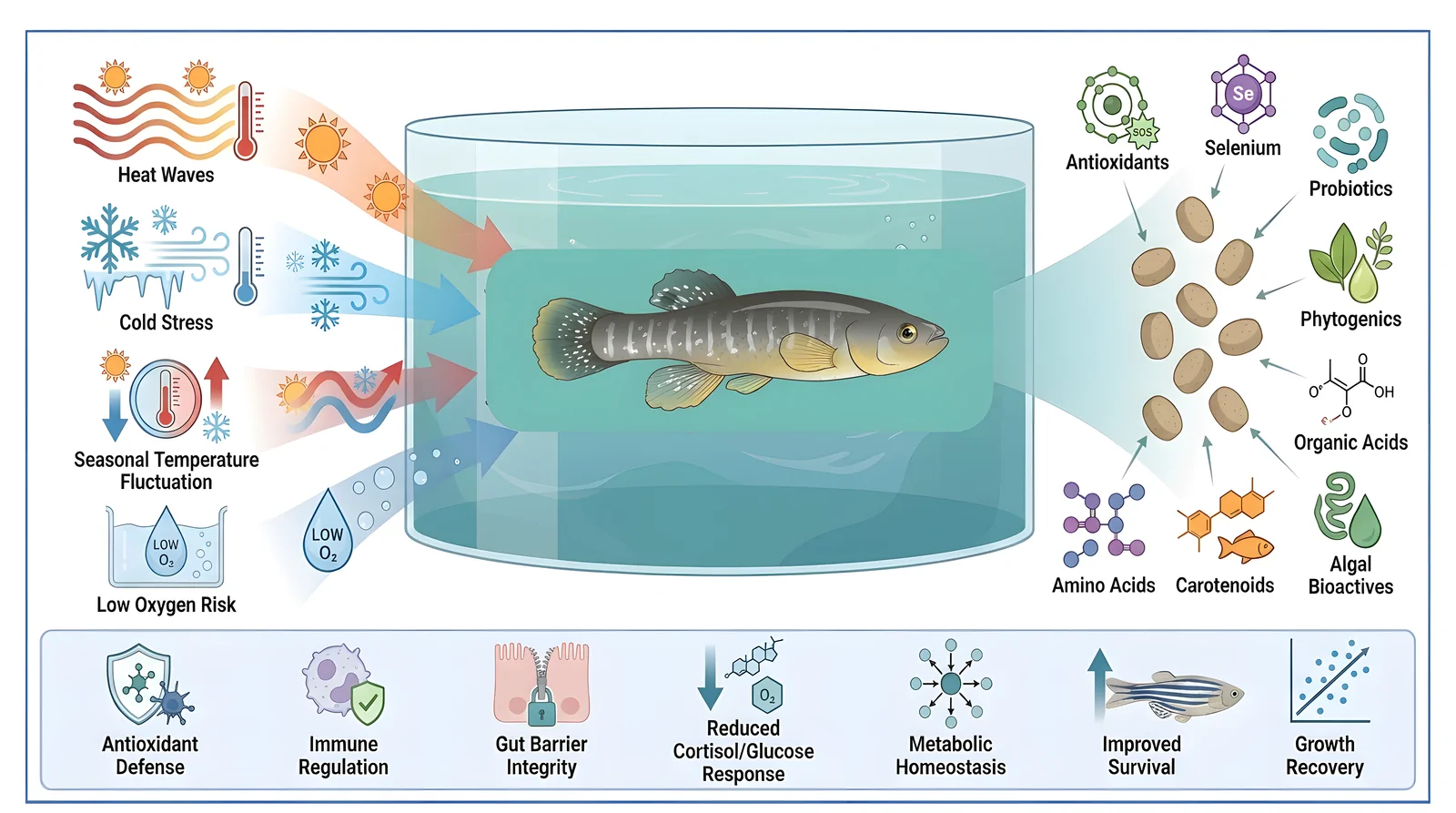
A conceptual synthesis of functional aquafeeds for climate-resilient finfish culture. Climate-related thermal stressors, including heat stress, cold stress, seasonal temperature instability and temperature fluctuation, can impair feed intake, nutrient utilization, endocrine regulation, oxidative balance, immunity, intestinal integrity and survival. Functional aquafeeds containing antioxidant micronutrients, selenium-based compounds, organic acids, probiotics, phytogenic additives, carotenoids, algal bioactives, functional amino acids and combined formulations may support resilience through coordinated regulation of antioxidant defense, cortisol/glucose responses, heat-shock proteins, innate and mucosal immunity, gut barrier function and metabolic homeostasis. This figure summarizes the overall conceptual basis of the review and should be interpreted as a synthesis framework rather than as a universal formulation model.

This systematic evidence map highlights the growing interest in functional aquafeeds as nutritional tools for improving thermal-stress resilience in cultured finfish. The evidence indicates that dietary interventions have been investigated not only for their effects on growth performance and feed utilization, but also for their capacity to modulate stress biomarkers, antioxidant defense, immune responses, gut health and metabolic homeostasis under heat, cold or sub-optimal temperature conditions. This reflects a broader transition in aquafeed research from conventional growth-oriented formulation toward resilience-oriented nutrition, in which dietary strategies are designed to support physiological stability under increasingly variable rearing environments. Based on the mapped evidence, the proposed conceptual framework indicates that functional aquafeeds may support climate resilience by linking dietary bioactive inputs with antioxidant, immune, gut and metabolic regulation under thermal stress (Figure 5).

The evidence base was not evenly distributed across species, functional-ingredient classes or thermal-stress models. Nile tilapia was strongly represented among the available studies, particularly in experiments evaluating sodium butyrate, organic selenium, *Bacillus* spp., propolis and essential oils under heat, cold or sub-optimal temperature conditions [36,47,51,56,72]. Other finfish species, including striped catfish and Wuchang bream, were included in studies evaluating selenium nanoparticles, riboflavin, emodin and vitamin C under elevated-temperature or stress-related conditions [58,62]. However, comparatively fewer studies were available for marine carnivorous species, salmonids, broodstock or long-term grow-out stages. This species imbalance limits the direct transferability of findings across cultured finfish groups, particularly because thermal tolerance, feeding habit, nutrient metabolism and immune regulation differ substantially among taxa.

Most studies focused on heat stress or cold stress, whereas repeated temperature fluctuation, seasonal transition and post-stress recovery were less frequently investigated. This is an important limitation because farmed fish are often exposed not only to constant high or low temperatures, but also to fluctuating and cumulative thermal disturbances. In addition, most studies measured a limited set of biomarkers rather than integrated response profiles. Growth, antioxidant enzymes and innate immune markers were commonly reported, whereas metabolomics, transcriptomics, microbiome analysis, intestinal barrier function and post-stress disease resistance were less consistently evaluated. Therefore, the current literature provides useful evidence that selected functional aquafeeds may support thermal-stress tolerance, but the mechanistic integration of immune, antioxidant, gut and metabolic responses remain incomplete.

### 4.2. Functional Aquafeeds as a Climate-Resilience Strategy

**Figure 6:**
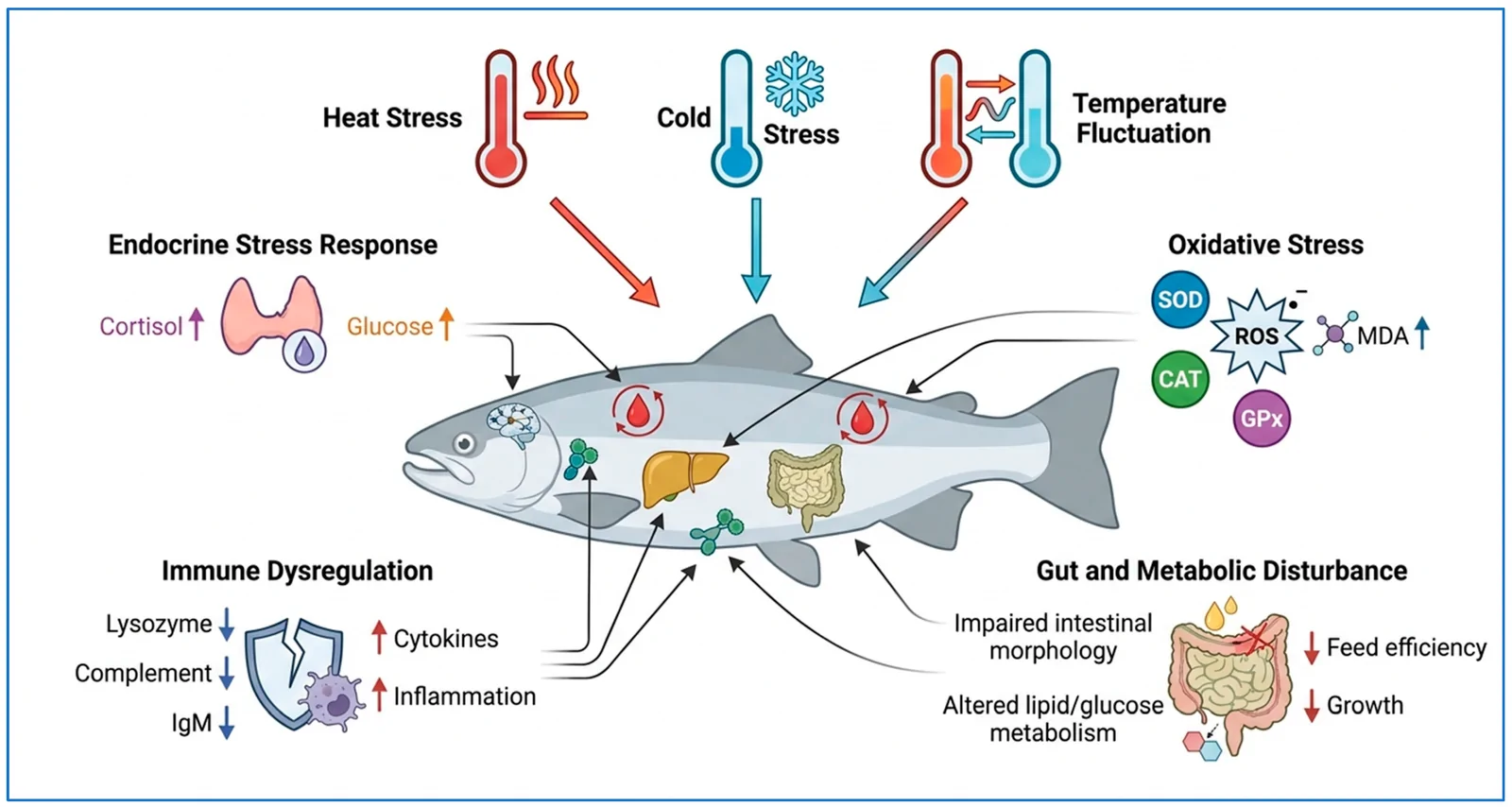
A mechanistic overview of thermal-stress responses in cultured finfish. Heat stress, cold stress and temperature fluctuation can activate endocrine stress pathways, increase cortisol and glucose mobilization, induce heat-shock protein responses, enhance reactive oxygen species production and alter antioxidant enzyme activity. When these responses are excessive or prolonged, thermal stress may lead to lipid peroxidation, immune suppression, intestinal barrier disruption, metabolic imbalance, impaired nutrient utilization and reduced growth or survival. The figure highlights why thermal-stress resilience should be interpreted as an integrated phenotype involving endocrine regulation, redox balance, immune function, gut integrity and metabolic homeostasis.

The findings suggest that functional aquafeeds may be considered promising tools for climate-resilient finfish culture, particularly when used within species-specific and stress-specific nutritional programs. Thermal stress affects fish at multiple biological levels, including endocrine stress regulation, oxidative balance, immune competence, intestinal function, nutrient metabolism and energy allocation. Because fish are ectothermic animals, changes in water temperature directly influence metabolic rate, feeding activity, digestion, oxygen demand and cellular homeostasis. Under thermal challenge, energy is often redirected from growth toward maintenance, stress defense, immune regulation and tissue repair. Therefore, nutritional strategies that help fish maintain antioxidant capacity, immune balance and metabolic stability may improve resilience even when growth enhancement is not always evident. The physiological effects of thermal stress are multi-systemic, involving endocrine stress activation, oxidative damage, immune disruption, intestinal impairment and altered energy allocation (Figure 6).

Several dietary interventions in the reviewed evidence produced coordinated improvements across multiple physiological domains. Sodium butyrate supplementation in Nile tilapia exposed to heat stress was associated with improved growth, intestinal morphology, immune response, antioxidant status, reduced cortisol and glucose, altered HSP70 expression and improved survival after heat exposure [36]. Organic selenium improved growth, serum biochemical indices, immune responses, antioxidative capacity and stress-related gene expression in Nile tilapia reared under sub-optimal temperature [47]. Selenium nanoparticles combined with riboflavin improved growth, antioxidant status, immune indicators and survival outcomes in striped catfish exposed to elevated temperature and arsenic stress [62]. These studies illustrate that functional aquafeeds may act through multiple interconnected pathways rather than through a single protective mechanism.

From a practical perspective, thermal-stress resilience should not be defined only as survival under extreme temperature. A more useful aquaculture definition should include maintenance of feed intake, feed efficiency, growth potential, immune competence, redox balance, gut integrity, metabolic stability and recovery capacity after stress exposure. Under this broader definition, functional aquafeeds can be viewed as nutritional conditioning tools. They may be used before predictable heatwaves, winter cold periods or seasonal transitions to prepare fish for stress, or after stress exposure to support recovery and reduce secondary disease risk.

### 4.3. Antioxidant Defense as a Central Mechanism of Thermal-Stress Tolerance

Antioxidant defense emerged as one of the most consistent response domains linking functional aquafeeds with thermal-stress resilience. Thermal stress can increase reactive oxygen species production, disturb mitochondrial function and promote lipid peroxidation. Therefore, dietary ingredients that enhance antioxidant enzyme activity or reduce oxidative damage are biologically plausible candidates for improving thermal tolerance. In the reviewed evidence, antioxidant-related benefits were reported for sodium butyrate, selenium, selenium nanoparticles, riboflavin, astaxanthin, propolis, bay laurel essential oil and algal-derived functional ingredients [36,47,51,56,60,62].

Improved antioxidant status was often reflected by increased superoxide dismutase, catalase, glutathione peroxidase, glutathione-related responses or total antioxidant capacity, together with reduced malondialdehyde. This combined pattern is more convincing than an increase in antioxidant enzyme activity alone. An isolated increase in antioxidant enzymes may represent either enhanced protective capacity or a compensatory response to persistent oxidative stress. In contrast, increased antioxidant capacity accompanied by reduced lipid peroxidation provides stronger evidence of improved redox protection. For example, sodium butyrate enhanced antioxidant enzyme activities and reduced malondialdehyde in Nile tilapia subjected to heat stress, while selenium nanoparticles and riboflavin reduced lipid peroxidation and improved antioxidant status in striped catfish under elevated-temperature stress [36,62].

Carotenoids and phytogenic additives also appear promising because of their direct and indirect antioxidant properties. Astaxanthin suppressed reactive oxygen species production induced by high-temperature stress and improved antioxidant defense in fish, suggesting that dietary carotenoids may help protect cellular structures during thermal challenge [60]. Propolis and bay laurel essential oil improved redox status and antioxidant defense in cold-stressed Nile tilapia, indicating that phytogenic additives may also support cold-stress tolerance through antioxidant and immunomodulatory pathways [51,56]. However, the effects of phytogenic compounds are likely to depend on source material, extraction method, active-compound concentration and dietary inclusion level. These factors should be carefully standardized in future studies.

**Figure 7:**
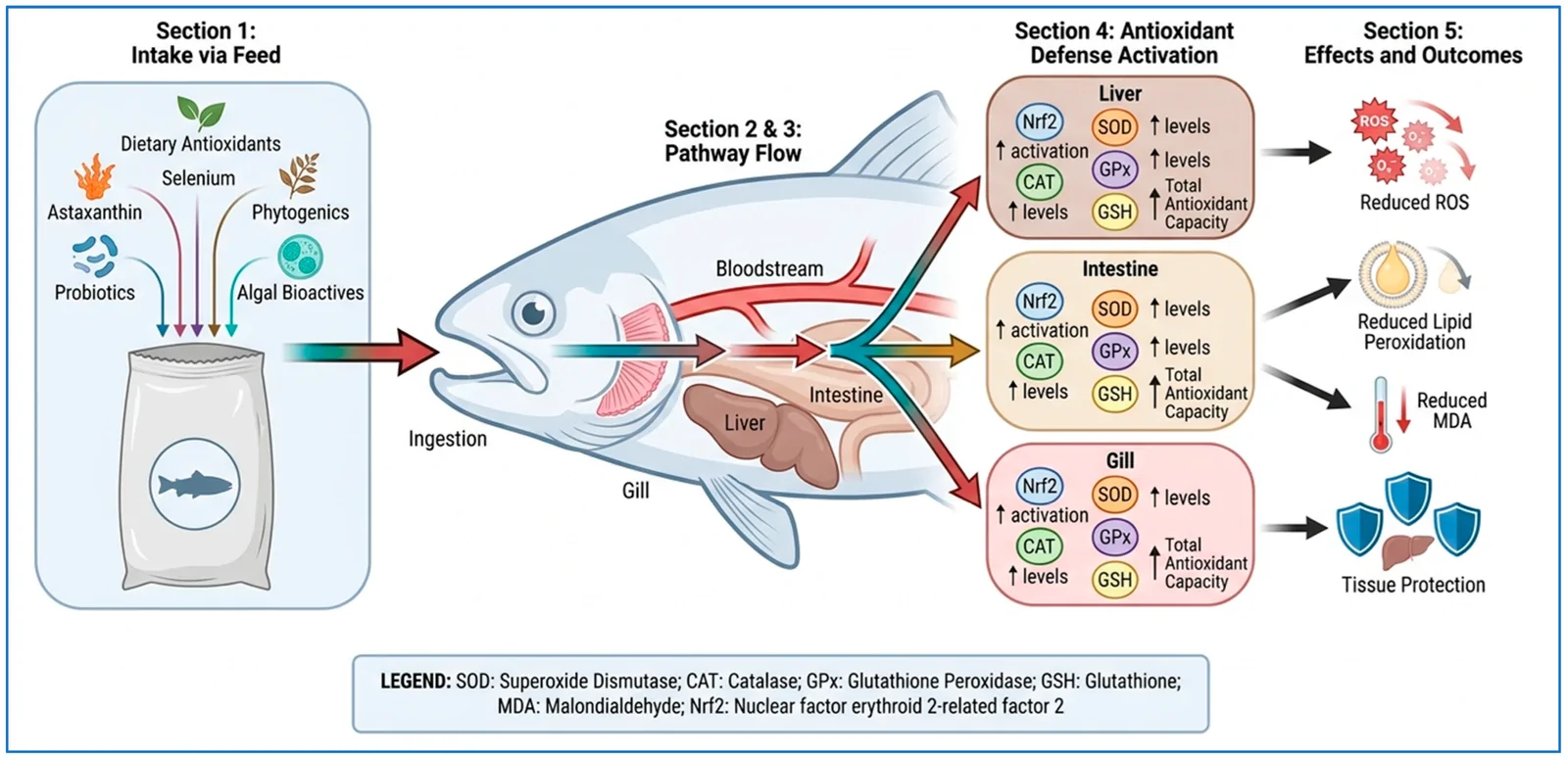
The proposed antioxidant-defense mechanism of functional aquafeeds under thermal stress. Thermal stress can increase mitochondrial reactive oxygen species production and promote oxidative damage in tissues such as the liver, intestine and gills. Functional feed ingredients, including selenium, vitamins, astaxanthin, phytogenic compounds, probiotics, organic acids and algal bioactives, may enhance antioxidant capacity by supporting SOD, CAT, GPx, GSH and total antioxidant capacity. A protective antioxidant response is most convincingly indicated when increased antioxidant activity is accompanied by reduced ROS, malondialdehyde and lipid peroxidation. This pathway may contribute to improved tissue protection, immune stability, metabolic balance and survival under heat or cold stress.

Overall, the antioxidant findings suggest that redox regulation is a central mechanism of functional-aquafeed-mediated thermal resilience. However, antioxidant biomarkers should be interpreted in an integrated manner. Future studies should combine antioxidant enzyme activity, oxidative-damage markers, stress hormones, immune indicators and performance outcomes to distinguish true physiological protection from compensatory stress responses. The proposed antioxidant-defense pathway through which functional aquafeeds may reduce thermal-stress damage is summarized in Figure 7.

### 4.4. Immune Regulation Under Thermal Stress

Thermal stress can impair immune competence, alter inflammatory responses and increase disease susceptibility in cultured fish. The reviewed studies suggest that functional aquafeeds may support immune stability during thermal challenge by enhancing innate immune defenses, regulating inflammatory responses and improving mucosal protection. Commonly reported immune indicators included lysozyme activity, complement activity, immunoglobulin levels, phagocytic activity, respiratory burst, myeloperoxidase activity, cytokine expression and antimicrobial defense-related genes.

Microbial-based additives were particularly relevant to immune regulation under thermal stress. Dietary *Bacillus* spp. supplementation mitigated heat-shock-related stress responses in Nile tilapia and influenced immune, antioxidant, histopathological and heat-shock protein gene-expression outcomes [72]. This suggests that probiotics may improve resilience through combined effects on immune readiness, oxidative status and tissue protection. Similarly, sodium butyrate improved phagocytic activity, lysozyme activity and immune response in heat-stressed Nile tilapia, indicating that gut-active functional ingredients can influence systemic immune competence during thermal challenge [36].

Phytogenic additives also showed immunomodulatory potential. Propolis improved growth, redox status and immune response in Nile tilapia subjected to cold stress, while bay laurel essential oil enhanced immunity and antioxidant defense in cold-stressed Nile tilapia at appropriate inclusion levels [51,56]. These findings support the view that phytobiotics may act through combined antioxidant, antimicrobial and anti-inflammatory mechanisms. However, immune stimulation should not be interpreted as universally beneficial. Excessive or prolonged inflammatory activation can increase metabolic cost and tissue damage. Therefore, the most desirable outcome is not simple immune upregulation, but balanced immune regulation.

This point is particularly important for interpreting cytokine responses. Increased expression of immune-related genes may indicate improved defense preparedness, whereas reduced pro-inflammatory cytokine expression may indicate attenuation of stress-induced inflammation. These responses are not necessarily contradictory. Functional aquafeeds may enhance basal defense capacity while preventing excessive inflammatory damage during thermal challenge. Therefore, immune outcomes should be interpreted in relation to stress severity, sampling time, tissue type, antioxidant status, gut health and survival outcomes.

### 4.5. Metabolic and Nutrient-Regulatory Responses and Energy Allocation

Metabolic homeostasis is a key component of thermal-stress resilience because temperature strongly affects energy demand, nutrient utilization and physiological maintenance. Under thermal stress, fish may increase glucose mobilization, alter lipid metabolism, change plasma protein status and redirect nutrients away from growth toward stress defense. Commonly reported metabolic and nutrient-regulatory indicators included total protein, triglycerides, cholesterol, ammonia, digestive-enzyme activity, nutrient-transport markers such as GLUT4, and genes related to lipid, glucose and nutrient metabolism. Cortisol and circulating glucose measured during or after thermal challenge were classified as endocrine stress-response indicators rather than metabolic outcomes.

Several dietary interventions produced complementary endocrine stress and metabolic or nutrient-regulatory responses under thermal challenge. Sodium butyrate reduced cortisol and circulating glucose in heat-stressed Nile tilapia, indicating moderation of the endocrine stress response, while its effects on total protein and intestinal morphology provided separate evidence of systemic and nutrient-related regulation [36]. Organic selenium improved serum biochemical indices and modulated transcription of stress-related genes in Nile tilapia under sub-optimal temperature [47]. Selenium nanoparticles and riboflavin reduced circulating glucose and cortisol, which were classified as stress-response outcomes, while also improving antioxidant and immune responses in striped catfish exposed to elevated temperature and arsenic stress [62]. These findings suggest that functional aquafeeds may moderate stress-related energy mobilization while supporting broader physiological adaptation under thermal stress.

Metabolic and nutrient-regulatory responses should not be interpreted from isolated changes in individual biochemical indicators. Although circulating glucose was classified within the endocrine and cellular-stress-response domain, its response pattern may provide cross-domain information regarding acute energy mobilization and recovery. Similarly, changes in triglycerides or cholesterol may reflect altered lipid mobilization, membrane adaptation or energy allocation. Therefore, metabolic homeostasis should be interpreted as the capacity to maintain coordinated energy balance, nutrient utilization and physiological function under thermal challenge.

The evidence also suggests that growth-related and metabolism-related genes can provide useful mechanistic insight. Bay laurel essential oil increased IGF-1 and GLUT4 expression at appropriate inclusion levels in cold-stressed Nile tilapia; IGF-1 was interpreted as a growth-regulatory indicator, whereas GLUT4 was classified as a metabolic and nutrient-regulatory marker [56]. Emodin and vitamin C altered biochemical parameters and HSP70 mRNA expression in Wuchang bream; the biochemical parameters were interpreted as metabolic responses, whereas HSP70 was classified within the cellular-stress-response domain [58]. However, gene-expression outcomes were highly variable across tissues, species and sampling points. Future studies should combine gene expression with plasma metabolites, enzyme activities and growth outcomes to better define metabolic resilience.

### 4.6. Gut Health as the Interface Between Diet and Systemic Resilience

**Figure 8:**
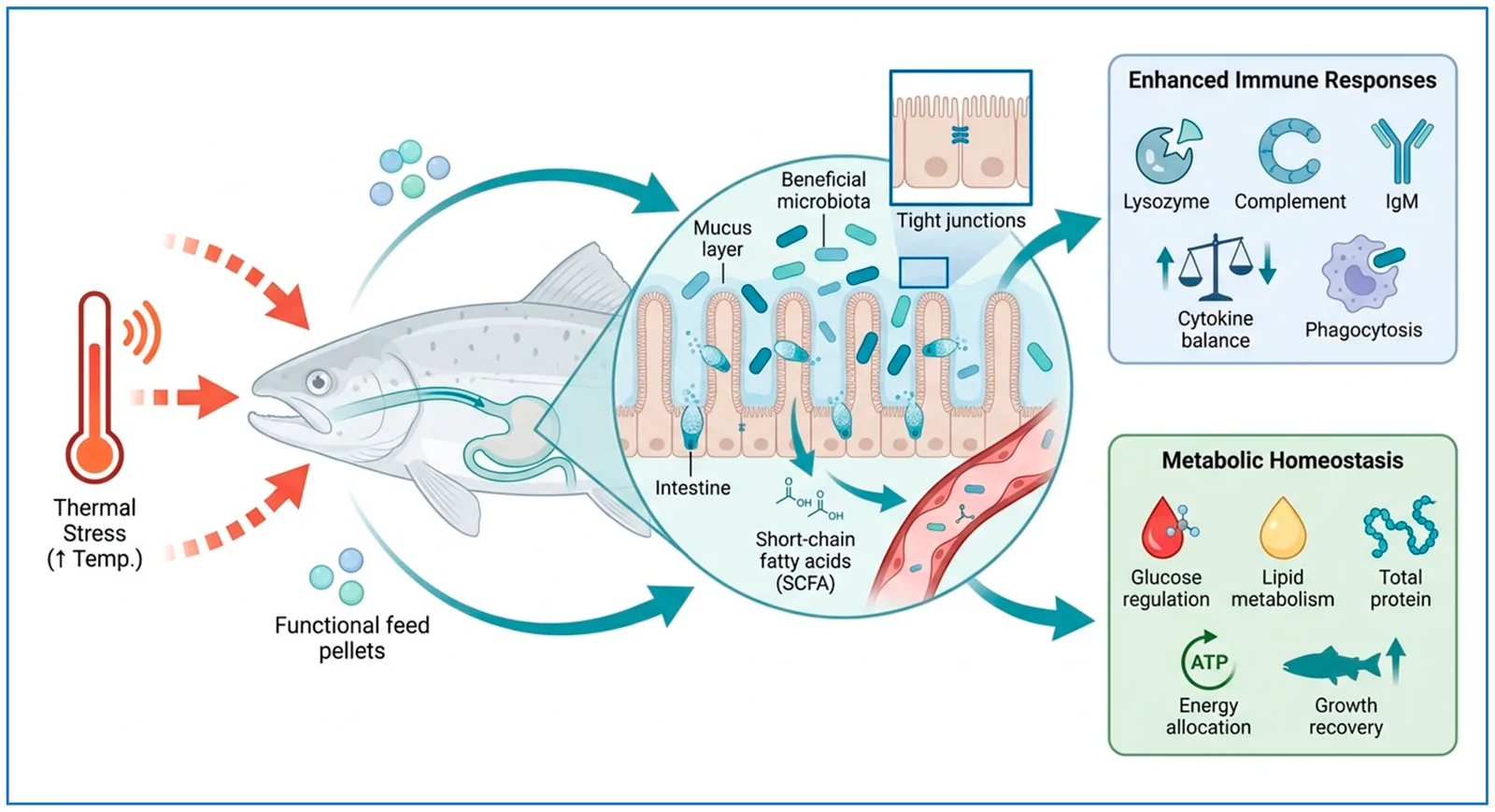
The gut–immune–metabolic axis linking functional aquafeeds with thermal-stress resilience. Functional aquafeeds may first act at the intestinal level by improving villus structure, goblet-cell abundance, mucus production, tight-junction integrity, digestive capacity and beneficial microbial communities. These local gut responses can support systemic resilience by improving nutrient absorption, mucosal and innate immunity, cytokine balance, antioxidant defense and metabolic homeostasis. This axis provides a mechanistic explanation for why gut-active additives such as sodium butyrate, probiotics, prebiotics, phytogenic compounds and algal bioactives may improve thermal-stress tolerance beyond direct growth promotion.

The intestine represents a major interface between functional aquafeeds and systemic stress resilience. Thermal stress can impair digestive function, intestinal morphology, mucosal barrier integrity and microbial balance, which may reduce nutrient absorption and increase inflammatory risk. Functional feed ingredients that improve intestinal morphology, goblet-cell abundance, mucin production, tight-junction integrity, digestive enzyme activity or beneficial microbiota may therefore enhance resilience indirectly by supporting nutrient acquisition and mucosal immune stability. The gut may function as a central interface through which functional aquafeeds influence systemic thermal-stress resilience by coordinating intestinal barrier function, mucosal immunity, microbial balance and nutrient metabolism (Figure 8).

In the reviewed evidence, gut-related outcomes were most frequently reported in studies evaluating organic acids, probiotics, phytogenic additives and algal-derived bioactives. Sodium butyrate improved intestinal histomorphology in Nile tilapia and was associated with enhanced immune response, antioxidant defense and survival after heat stress [36]. *Bacillus* spp. supplementation influenced histopathological status and stress-protective responses in heat-shocked Nile tilapia, supporting the potential role of probiotics in maintaining tissue integrity during thermal challenge [72]. These findings suggest that gut-active functional ingredients may help preserve intestinal function when thermal stress compromises digestion and mucosal immunity.

Despite these promising findings, gut-mediated thermal resilience remains insufficiently resolved. Many studies reported intestinal morphology without microbiome analysis, or immune biomarkers without barrier-function markers. Others measured systemic responses but did not evaluate the intestine. This limits understanding of how local gut responses translate into whole-body resilience. Future studies should integrate intestinal histology, digestive enzymes, mucosal immunity, tight-junction gene expression, gut microbiota and metabolite profiling. Such integrated designs would clarify whether improved growth or survival under thermal stress is mediated through improved digestion, barrier protection, microbial modulation or systemic immune–metabolic regulation.

### 4.7. Ingredient-Specific Interpretation

Ingredient-specific interpretation followed the hierarchical framework defined in Section 2.7, which separated intervention identity and source from biological function. Chemically defined nutrients and metabolites included vitamins, minerals and trace elements, amino acids, lipids, organic acids and carotenoids. Microbial preparations, natural-product-derived bioactives and algal preparations were classified according to source, whereas multicomponent interventions were treated as combination formulations. Antioxidant, immunomodulatory, gut-protective and metabolic effects were interpreted as overlapping mechanisms rather than ingredient categories.

Within the chemically defined nutrient and metabolite class, functional amino acids and related compounds may support thermal-stress resilience through osmoregulation, nitrogen metabolism, neurotransmission, antioxidant defense, protein turnover and immune-cell metabolism. Although amino-acid-based interventions were less consistently represented than vitamins, minerals or natural-product-derived bioactives, they remain important candidates for resilience-oriented feed formulation because thermal stress increases the demand for cellular repair, antioxidant substrates and metabolic regulation.

Vitamins, minerals and trace elements, including vitamin C, vitamin E, selenium and copper, are particularly relevant when thermal stress induces oxidative damage. Selenium-based interventions were among the clearer examples in the reviewed evidence. Organic selenium improved growth, immunity, antioxidative capacity and stress-related gene expression in Nile tilapia under sub-optimal temperature, whereas selenium nanoparticles combined with riboflavin enhanced antioxidant and immune outcomes under elevated-temperature stress in striped catfish [47,62]. These findings support the role of micronutrients in maintaining redox balance and immune competence under thermal stress. However, micronutrients have narrow safe and effective ranges; therefore, dose–response studies are essential. Astaxanthin, classified within the carotenoid subclass, also improved high-temperature stress resistance through antioxidant-related mechanisms [60].

Natural-product-derived bioactives, including botanical extracts, essential oils, propolis and fungal-derived products, may act through antioxidant, anti-inflammatory, antimicrobial and appetite-modulating mechanisms. Propolis and bay laurel essential oil improved growth, redox status and immune responses under cold-stress conditions in Nile tilapia [51,56]. Nevertheless, these interventions are difficult to compare because their biological effects depend on source material, extraction method, active-compound concentration and dietary inclusion level. Future studies should provide chemical characterization to improve reproducibility and formulation value.

Microbial preparations, including single- and multi-strain probiotics, may improve thermal resilience through modulation of gut microbiota, mucosal immunity, digestive function and pathogen resistance. The positive effects of *Bacillus* spp. under heat shock suggest that probiotics may buffer thermal stress by improving immune and antioxidant responses while reducing tissue damage [72]. Organic acids such as sodium butyrate were classified separately within chemically defined nutrients and metabolites and may support gut-mediated resilience by improving intestinal morphology and immune responses under heat stress [36].

Algal preparations, including single-source *Chlorella* and *Spirulina* interventions, represent promising but still limited evidence categories. Combination formulations, such as selenium nanoparticles–riboflavin, multicomponent supplement blends and *Spirulina*–coenzyme Q10, were classified separately because their effects could not be attributed to a single component. Their future value will depend on stronger evidence linking composition, dose and component interactions with physiological outcomes under defined thermal-stress models.

### 4.8. Practical Implications for Climate-Resilient Aquafeed Formulation

The findings have practical implications for climate-resilient aquafeed development, but these implications should be treated as a research-guidance framework rather than direct universal formulation recommendations. Functional aquafeeds should not be viewed only as continuous growth-promoting diets, but also as strategic feeds that may be applied during predictable high-risk periods. For example, antioxidant- and immune-supportive formulations may be useful before summer heatwaves, winter cold periods or seasonal temperature transitions. During thermal stress, highly digestible diets enriched with functional nutrients may help reduce metabolic burden. After stress exposure, recovery-oriented diets may support gut repair, antioxidant restoration, immune stabilization and metabolic rebalancing.

**Figure 9:**
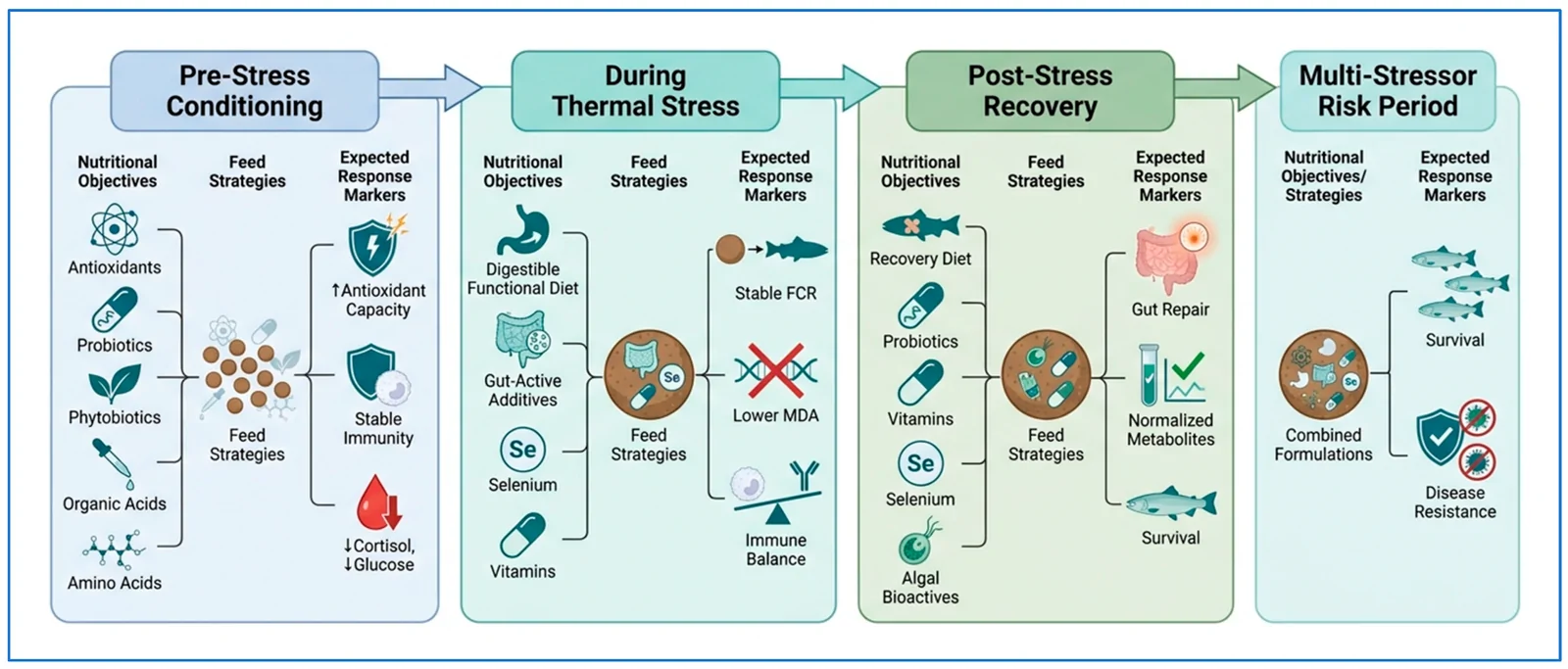
A translational framework for applying functional aquafeeds in climate-resilient finfish culture. Functional feeds may be applied strategically across four practical phases. During pre-stress conditioning, diets may aim to enhance antioxidant capacity, immune readiness, gut integrity and metabolic reserves before predictable heatwaves, cold periods or seasonal transitions. During thermal stress, highly digestible functional diets may help maintain feed intake, redox balance and immune competence while limiting cortisol, glucose and oxidative-damage responses. During post-stress recovery, recovery-oriented diets may support intestinal repair, immune stabilization, metabolic rebalancing and survival. During multi-stressor periods, combined antioxidant–immune–gut formulations may help buffer thermal, water-quality and pathogen-related risks. This framework should be interpreted as a research-guidance model rather than universal formulation guidance.

A practical feeding framework may include three stages: pre-stress conditioning, stress-period support and post-stress recovery. Accordingly, functional aquafeeds can be organized into a practical climate-resilience framework consisting of pre-stress conditioning, stress-period support, post-stress recovery and multi-stressor risk management phases (Figure 9).

Pre-stress conditioning aims to increase physiological preparedness by strengthening antioxidant capacity, mucosal immunity and metabolic reserves. Stress-period support aims to maintain feed intake, digestion and cellular protection while minimizing oxidative and inflammatory damage. Post-stress recovery aims to restore intestinal integrity, immune balance and growth performance. This approach is more realistic than expecting a single additive to solve all thermal-stress problems. A translational framework for applying these strategies in climate-resilient aquafeed programs is summarized in Table 6.

However, practical formulation must remain species-specific and dose-specific. A functional ingredient that benefits Nile tilapia under heat stress may not produce the same effect in cold-water salmonids, marine carnivores or herbivorous species. Feeding habit, basal diet composition, thermal preference, life stage and culture environment all influence response to supplementation. Therefore, functional-aquafeed formulation should be based on species-specific nutrient requirements, predictable environmental risk and clearly defined physiological targets.

### 4.9. Methodological Limitations of the Current Evidence

Several limitations were identified in the current evidence. First, thermal-stress protocols were not standardized. Studies differed in control temperature, stress temperature, exposure duration, temperature-shift rate, acclimation period, sampling time and recovery assessment. These differences make it difficult to compare dietary interventions directly across studies. Future studies should report thermal protocols in sufficient detail and justify stress temperatures according to species-specific thermal ranges.

Second, many studies relied on limited biomarker panels. Although antioxidant enzymes, cortisol, glucose and immune markers are useful, they provide only partial insight into resilience. Thermal-stress resilience is a multisystem phenotype involving growth, feed intake, endocrine stress response, redox balance, immune regulation, gut integrity, nutrient metabolism and recovery capacity. Studies that measure only one or two domains may overestimate or underestimate the functional value of a dietary intervention.

Third, dose–response designs were not consistently used. Single-dose supplementation can identify potential effects, but it cannot determine optimal, marginal or excessive inclusion levels. This is particularly important for selenium, essential oils, phytogenic extracts and immunostimulants, where excessive inclusion may produce neutral or negative effects. Fourth, the reporting of randomization, blinding and experimental unit was often incomplete. In aquaculture feeding trials, the tank rather than the individual fish is usually the appropriate experimental unit when diet is administered at tank level. Therefore, future studies should clearly report tank number, fish number per tank, random tank allocation, whether tanks or individual fish were used in the statistical model and how subsampling within tanks was handled. Failure to clearly define the experimental unit may increase the risk of pseudoreplication and overinterpretation.

The identified experimental-unit concerns also affected the confidence assigned to specific outcomes. When dietary treatments were allocated at tank level, fish sampled from the same tank represented subsamples rather than independent treatment replicates. Treating these fish as independent observations may underestimate variance and overstate statistical precision. In the present evidence map, this concern primarily affected biochemical, immune, histological and molecular endpoints. Accordingly, these outcomes were interpreted as supportive or hypothesis-generating rather than confirmatory, whereas independently analyzed tank-level growth and feed-utilization outcomes from the same studies were considered separately. Study-specific affected outcomes and their consequences for interpretation are detailed in Supplementary Table S4C.

A further limitation arises from the substantial species and design heterogeneity of the included evidence. The available studies were strongly concentrated in Nile tilapia, whereas marine carnivores, salmonids, broodstock and long-term grow-out stages were comparatively under-represented. Species differed in thermal preference, feeding habit, nutrient metabolism and stress physiology, while the studies also varied in dietary intervention, supplementation level, feeding duration, thermal-challenge protocol, sampled tissue and outcome definition. These differences prevented a meaningful quantitative synthesis. Consequently, no pooled effect sizes, confidence intervals, statistical heterogeneity estimates, subgroup analyses or quantitative comparisons among intervention classes were generated. The evidence map therefore identifies response directions, mechanistic patterns and research concentrations but cannot determine the magnitude or precision of additive-specific effects or establish a quantitative ranking of interventions. Apparent differences among dietary strategies may partly reflect species, protocol and outcome heterogeneity rather than true differences in efficacy. The findings should therefore be interpreted as qualitative and mechanistic evidence rather than as pooled efficacy estimates or universal formulation recommendations.

An additional limitation concerns evidence retrieval. The review did not systematically search dedicated gray-literature databases or solicit unpublished datasets from domain experts, and eligibility was restricted to English-language peer-reviewed articles. Consequently, non-English, unpublished, null or negative findings may be under-represented, potentially increasing the apparent predominance of beneficial dietary responses. The evidence map should therefore be interpreted as a synthesis of the accessible peer-reviewed English-language literature rather than a complete inventory of all conducted studies.

Finally, relatively few studies evaluated post-stress recovery, repeated temperature fluctuation or combined thermal-stress and disease-challenge models. This is a major gap because farmed fish are often exposed to multiple stressors under commercial conditions. Thermal stress may increase disease susceptibility, reduce appetite, alter gut microbiota and impair recovery. Therefore, studies that integrate thermal challenge, recovery monitoring and pathogen resistance are needed to determine whether functional aquafeeds provide meaningful farm-level resilience.

### 4.10. Future Research Priorities

Future research should move from single-marker evaluation toward integrated resilience phenotyping. At minimum, studies should combine growth and feed-utilization outcomes with stress biomarkers, antioxidant status, immune indicators, gut-health markers and metabolic responses. A core biomarker panel could include weight gain, specific growth rate, feed conversion ratio, survival, cortisol, glucose, SOD, CAT, GPx, MDA, lysozyme, complement activity, IgM, key cytokines, intestinal morphology and selected metabolic indicators. When possible, transcriptomics, metabolomics and microbiome analysis should be added to identify mechanistic pathways and biomarker signatures of resilience.

Future research should move beyond descriptive microbiome profiling toward microbiome-informed dietary interventions. Probiotics, prebiotics, synbiotics, postbiotics and diet–microbiome combinations should be selected for their ability to maintain microbial stability, short-chain fatty acid production, mucosal integrity and immune–metabolic regulation during thermal stress. Artificial intelligence and machine learning may support context-specific aquafeed formulation by integrating species, life stage, diet composition, functional ingredients, water temperature, dissolved oxygen, feeding behavior, growth and physiological biomarkers. In precision aquaculture, sensors, automated feeding, computer vision and decision-support models could adjust feeding rate, timing and additive application according to temperature, appetite, behavior and water quality. However, standardized datasets, transparent models, external validation and biological confirmation are required to ensure improvements in fish welfare, thermal resilience and production performance.

Thermal-stress models also require greater standardization. Future studies should clearly report optimal temperature, stress temperature, temperature-shift rate, exposure duration, recovery period and sampling time. Studies should distinguish between acute heat shock, chronic high-temperature exposure, cold shock, chronic low-temperature exposure and repeated temperature fluctuation. These models may produce different physiological responses and should not be interpreted as equivalent.

More attention should be given to under-represented species and production stages. Research remains heavily concentrated in Nile tilapia, while marine carnivorous fish, salmonids, yellowtail, groupers, olive flounder, sea bass and sea bream require further investigation. Broodstock, larvae and long-term grow-out stages are also under-represented. Because nutritional requirements and stress tolerance differ across life stages, functional aquafeeds should be validated across production phases.

**Figure 10:**
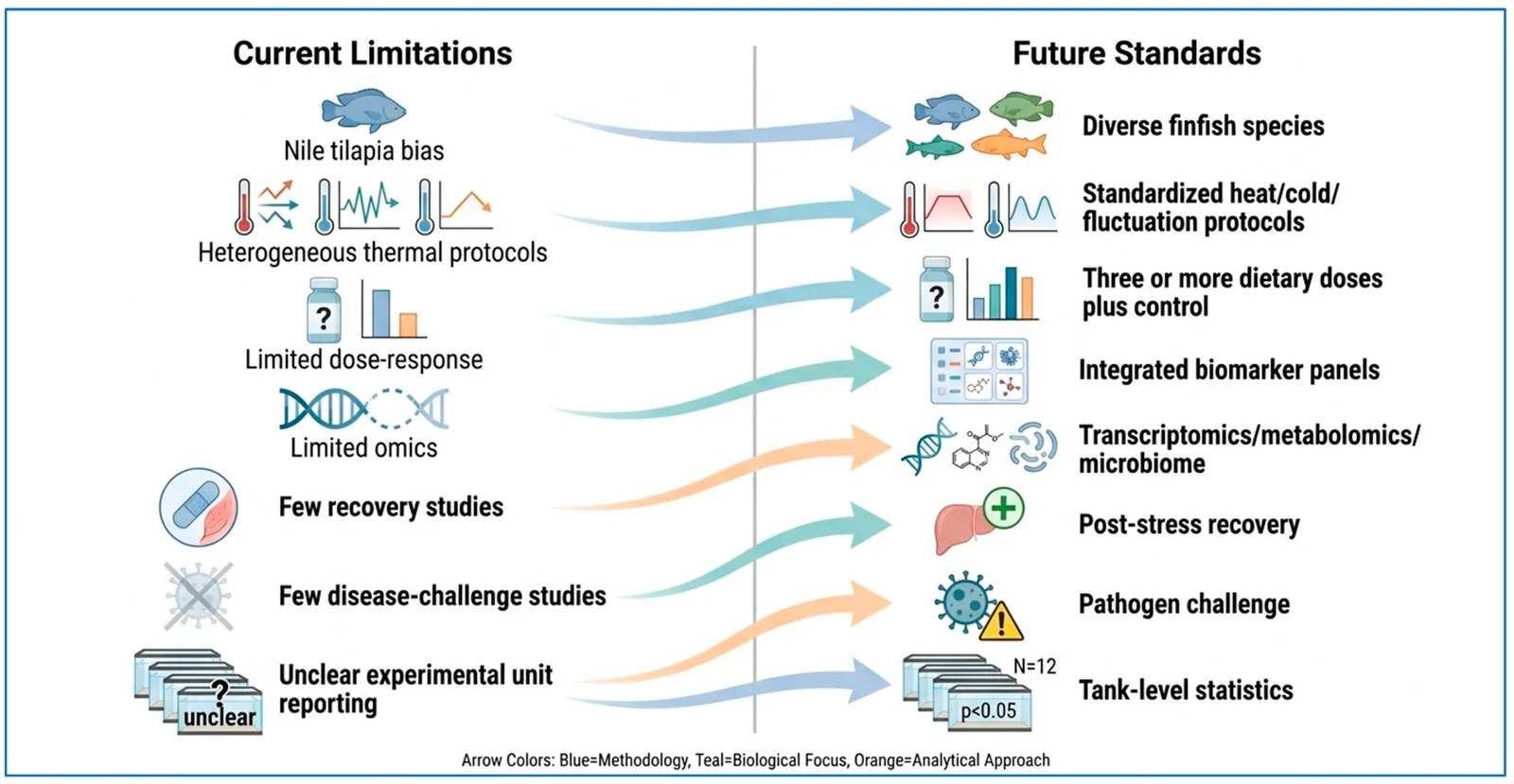
Evidence gaps and a future research roadmap for climate-resilient functional-aquafeed studies. The current evidence base is limited by species imbalance; concentration of studies in Nile tilapia; heterogeneous thermal-stress protocols; incomplete dose–response testing; limited post-stress recovery assessment; few combined thermal-stress and disease-challenge models; limited integration of transcriptomics, metabolomics and microbiome analysis; and incomplete reporting of randomization, blinding and experimental units. Future studies should include diverse finfish species; standardized heat, cold and temperature-fluctuation models; at least three supplementation levels plus a control; clear tank-level replication; integrated biomarker panels; recovery sampling; pathogen-challenge validation; and long-term farm-relevant testing. Addressing these priorities will improve comparability, mechanistic interpretation and practical translation of functional aquafeeds for climate-resilient aquaculture. Future research should also integrate microbiome-informed dietary strategies, artificial intelligence-assisted feed formulation and sensor-based precision feeding to support adaptive nutritional management under changing thermal conditions.

Future studies should assess practical feeding strategies, including pre-stress, seasonal, recovery and combined functional diets. Multi-ingredient formulations may better target antioxidant, immune, gut and metabolic pathways, but their additive, synergistic and antagonistic effects must be distinguished. Key evidence gaps and methodological priorities are summarized in Figure 10.

## 5. Conclusions

Overall, the available evidence suggests that selected functional aquafeeds may improve thermal-stress resilience in cultured finfish, mainly through antioxidant defense, immune regulation, gut integrity, stress-biomarker modulation and metabolic stability. Sodium butyrate, selenium-based supplements, carotenoids, phytogenic additives and microbial-based additives showed promising effects under heat, cold or sub-optimal temperature conditions. However, responses varied according to species, dose, basal diet, stress model, exposure duration and measured endpoint. Therefore, the conclusions should be interpreted as a structured evidence-map synthesis rather than as universal formulation guidance.

The central message from this review is that climate-resilient aquafeeds should be designed not only to maintain growth, but also to support physiological stability under environmental stress. Thermal-stress resilience is a coordinated phenotype involving endocrine regulation, redox balance, immune competence, intestinal function and energy metabolism. To strengthen translation into practical aquafeed formulation, future studies should use standardized thermal-challenge protocols, dose–response designs, clear tank-level experimental-unit reporting, integrated biomarker panels, multiomics and microbiome-informed approaches, artificial intelligence-assisted formulation, precision aquaculture tools, post-stress recovery assessment and long-term validation under realistic farming conditions. Greater attention to under-represented marine carnivorous species, salmonids, broodstock and grow-out stages will be especially important for determining whether promising findings from current model species can be generalized across finfish aquaculture.

## Figures and Tables

**Table 1:** Class-level evidence synthesis of functional-aquafeed interventions evaluated for thermal-stress resilience in cultured finfish.

Primary Intervention Class	Representative Interventions	Thermal Contexts	Main Findings	Confidence and Limitations	References
Chemically defined nutrients and metabolites (*n* = 11)	Sodium butyrate; selenium sources; vitamins C/E; copper; astaxanthin; taurine; alpha-lipoic acid; dietary lipid levels and sources.	Heat, cold, sub-optimal temperature and multi-stressor models.	Mostly protective: improved growth or survival, antioxidant and immune status, stress markers, and gut or tissue condition. Lipid effects varied by level and source.	Moderate; the largest evidence cluster, but interventions and protocols were diverse, and some mechanistic outcomes had experimental-unit concerns.	[36,47,48,49,52,54,60,61,63,68,69]
Microbial preparations (*n* = 2)	Mixed *Bacillus* spp. and a probiotic mixture.	Acute heat shock and higher-temperature rearing.	Protective effects support a gut–immune–redox mechanism.	Low to moderate; only two studies were available, with some analytical-unit concerns.	[59,72]
Natural-product-derived bioactives (*n* = 9)	Mushroom, curcumin, propolis, *Pelargonium*, essential oils, plant extracts and tea polyphenols.	Heat and cold challenges, heat-resistance tests and critical thermal minimum models.	Mostly protective and dose-dependent effects on growth or survival, redox balance, immunity, inflammation and metabolic indicators.	Low to moderate; substantial chemical and dose heterogeneity, with experimental-unit concerns in several studies.	[46,50,51,55,56,64,65,66,70]
Algal preparations (*n* = 2)	*Chlorella vulgaris* powder and nanoparticles; dietary *Spirulina platensis*.	Cold/hypoxia and heat-stress models	Protective effects on growth, antioxidant status, immunity and hepatic or tissue condition.	Emerging evidence from only two heterogeneous studies with incomplete design reporting.	[53,71]
Combination formulations (*n* = 4)	Emodin–vitamin C, SeNPs–riboflavin, supplement blends, and *Spirulina*–CoQ10 nanoemulsion.	Heat, heatwave, multi-stressor and post-heat bacterial-challenge models.	Mostly protective multisystem effects; one study was primarily mechanistic. Component-specific effects and synergy could not be separated.	Emerging to moderate; formulations were heterogeneous, and all four studies had experimental-unit concerns affecting some outcomes.	[57,58,62,67]

Note: Classes are mutually exclusive, although each study could contribute to several outcome domains. Confidence represents a qualitative body-of-evidence judgment based primarily on the number of independent studies, consistency of response direction, methodological limitations and risk of bias, experimental-unit validity, species and thermal-model relevance, protocol clarity and dose–response support. Concurrent improvement across multiple outcome domains was interpreted as biological or translational coherence and did not independently increase the confidence category. Outcomes affected by experimental-unit concerns were treated as supportive evidence. Representative study extraction is provided in Supplementary Table S3, while complete design, diet and thermal-protocol details are provided in Supplementary Table S3A,B and Supplementary File S1.

**Table 2:** Synthesis of thermal-stress models and implications for evidence interpretation.

Thermal-Stress Context	Studies/Examples in Current Evidence Map	Response Domains Captured	Synthesis Value	Main Limitation for Cross-Study Comparison
Heat stress or heat shock	Butyrate, *Bacillus*, and *Spirulina*–CoQ10 in tilapia, plus astaxanthin under heat stress.	Growth, survival, stress, antioxidant, immune, HSP, and tissue responses.	Strongest evidence supports antioxidant–immune–gut–metabolic protection.	Variable stress temperatures, exposure durations, sampling times and recovery assessments.
Cold stress	Propolis and bay laurel essential oil in Nile tilapia.	Growth, redox, immunity, inflammation, IGF-1, and GLUT4.	Functional aquafeeds may enhance both cold and heat tolerance.	Evidence is species-limited and mainly focused on Nile tilapia.
Sub-optimal temperature	Organic selenium in Nile tilapia.	Growth, serum biochemistry, immunity, antioxidant status, and stress genes.	Useful model for chronic non-optimal rearing conditions relevant to seasonal stress.	Needs clearer distinction from acute cold shock and chronic cold stress.
Elevated temperature with additional stressor	SeNPs + riboflavin under elevated temperature plus arsenic in striped catfish.	Growth, stress, antioxidant, immune, HSP70, and survival responses.	Realistic multi-stressor conditions highlight nutritional buffering.	Difficult to separate thermal-specific effects from chemical-stress effects.
Temperature fluctuation and recovery models	Limited representation in the evidence map.	Rarely integrated across growth, stress, gut, immune, and metabolic responses.	Highly relevant to farm conditions and climate variability.	Major evidence gap; future studies should assess repeated fluctuations and recovery.

Note: This table provides a synthesis of the physiological and interpretive implications of different thermal-stress models. Study-level quantitative protocol characteristics and operational classifications, including reference temperature, stress temperature, ΔT, temperature-change pattern, exposure duration and recovery assessment, are provided in Supplementary Table S3B.

**Table 3:** Mechanistic synthesis by outcome domain.

Outcome Domain	Common Indicators Extracted	Dominant Evidence Pattern	Ingredient Classes Most Often Linked	Interpretive Synthesis	References
Growth performance and survival	WG, SGR, FCR/FE, survival, relative survival after challenge.	Generally protective, but neutral under severe stress or sub-optimal doses.	Sodium butyrate, selenium, SeNPs–riboflavin, propolis, essential oils, and *Spirulina* products.	Growth benefits likely reflect reduced stress and improved physiological stability.	[36,47,51,57,62]
Endocrine and cellular-stress response	Cortisol, glucose, lactate, HSP70/HSP90, and stress-related genes.	Supplementation often moderated cortisol, glucose, and HSP responses.	Sodium butyrate, SeNPs–riboflavin, organic selenium, *Bacillus* spp., and emodin–vitamin C.	Cortisol and glucose reflect systemic stress, while HSPs indicate cellular stress.	[36,47,58,62,72]
Antioxidant defense and oxidative damage	SOD, CAT, GPx, GSH, T-AOC, ROS, MDA/lipid peroxidation.	Protection involved higher antioxidant enzymes and lower MDA or lipid peroxidation.	Butyrate, selenium, SeNPs–riboflavin, astaxanthin, propolis, bay laurel oil, and *Spirulina*.	Redox regulation supports thermal resilience but requires oxidative-damage markers.	[36,47,51,56,60,62]
Innate, mucosal and inflammatory immunity	Innate immune, inflammatory, antimicrobial, and mucosal markers.	Functional diets often enhanced innate immunity or moderated inflammation.	*Bacillus* spp.; sodium butyrate; propolis; bay laurel oil; SeNPs + riboflavin; *Spirulina*-based products.	Thermal resilience depends on balanced immune regulation, not simple stimulation.	[36,51,56,62,71,72]
Metabolic and nutrient-regulatory responses	Protein, lipid, ammonia, digestive, glycogen, GLUT4, gene, and metabolomic markers.	Dietary interventions modulated biochemical, digestive, and energy-metabolism responses.	Sodium butyrate, organic selenium, SeNPs–riboflavin, bay laurel oil, and emodin–vitamin C.	Metabolic homeostasis required coordinated responses across multiple indicators.	[36,47,56,58,62]
Gut health and tissue integrity	Intestinal morphology, goblet cells, digestion, absorption, and tissue condition.	Gut-active and combined bioactives mainly improved gut and tissue protection.	Sodium butyrate; *Bacillus* spp.; *Spirulina*–CoQ10; phytogenic products.	The gut supports thermal resilience through digestion, barrier protection, and mucosal immunity.	[36,57,72]
Disease resistance after dietary or thermal conditioning	Cumulative mortality, post-challenge survival, relative percent survival.	Evidence is limited but promising, with three multi-stressor pathogen-challenge studies.	Dietary copper; SeNPs + riboflavin; *Spirulina*–CoQ10.	Post-stress disease resistance is important but underexplored and methodologically limited.	[57,62,63]

Note: WG, weight gain; SGR, specific growth rate; FCR, feed conversion ratio; FE, feed efficiency; HSP, heat-shock protein; SOD, superoxide dismutase; CAT, catalase; GPx, glutathione peroxidase; MDA, malondialdehyde. Outcome variables were assigned to one primary domain for evidence-map counting. Cortisol and circulating glucose measured during or after thermal challenge were classified as endocrine stress indicators, whereas tissue glucose transport, glycogen and glucose-metabolism pathways were classified as metabolic indicators. IGF-1 was treated as a growth-regulatory indicator, and hemoglobin, hematocrit and blood-cell counts were treated as supportive hematological indicators. Metabolic homeostasis was not inferred from a single biomarker.

**Table 4:** Cross-domain evidence profile of representative functional-aquafeed interventions under thermal stress.

Intervention	Growth/Survival	Stress Response	Antioxidant Defense	Immune Response	Metabolic Regulation	Gut/Tissue Integrity	Disease Resistance	Main Limitation
Sodium butyrate [36]	Higher growth, FE, and heat survival	Lower cortisol/glucose; HSP70 modulated	Higher SOD, CAT, GPx; lower MDA	Higher lysozyme and phagocytosis	Higher total protein	Better gut morphology and goblet cells	NR	One Nile tilapia study
Single-component selenium [47,68]	Higher growth at sub-optimal temperature	Stress genes modulated	Higher antioxidant capacity	Higher immune indices	Better serum biochemistry	Nano-Se: less gut damage; microbiota shift	NR	Forms, species, and protocols vary
Dietary lipid strategies [48,49]	Dose/source-dependent growth and cold tolerance	Not consistently assessed	Limited evidence	Limited evidence	Energy supply and membrane adaptation	Liver condition assessed	NR	Only Nile tilapia cold models
Astaxanthin/taurine [60,61]	Growth or thermal protection	Stress/apoptosis responses modulated	Lower ROS or better protection	Astaxanthin: higher innate immunity	Taurine: supports Ca^2+^ homeostasis	Inconsistent evidence	NR	One study each; opposite stress directions
*Bacillus* spp./probiotic mixture [59,72]	Growth protection	HSP70, stress, and apoptosis modulated	Higher antioxidant status	Higher hemato-immune responses	Limited evidence	Histological protection; limited gut data	NR	Two studies; unit-of-analysis concerns
Natural-product bioactives [46,50,51,55,56,64,65,66,70]	Mostly beneficial; dose-dependent	Variable stress responses	Generally better redox status	Frequent immune/inflammatory modulation	Selected effects on GLUT4, enzymes, and lipids	Limited barrier/microbiota data	NR	High chemical, dose, and protocol heterogeneity
*Chlorella*/*Spirulina* [53,71]	Better performance under cold/hypoxia or heat	Limited evidence	Higher antioxidant responses	Higher immunity, especially with Spirulina	Supportive liver/serum markers	Limited gut/barrier data	NR	Two different species and models
SeNPs + riboflavin [62]	Higher growth, FE, PER, and SGR	Lower cortisol, glucose, and HSP70	Lower lipid peroxidation; higher antioxidants	Higher NBT, Ig, MPO, and globulin	Higher tissue vitamin C	Tissue protection	Higher survival; lower mortality	Multi-stressor model; fish-level unit concerns
Emodin + vitamin C/blend [58,67]	Mixed; mainly mechanistic	HSP70 and cellular responses assessed	Oxidative markers assessed	Limited or formula-specific	Metabolic and molecular effects	Inconsistent evidence	NR	Individual effects and synergy unresolved
*Spirulina*–CoQ10 [57]	Higher growth and survival	Limited endocrine evidence	Higher antioxidant biomarkers	Higher immune biomarkers	Supportive biochemistry	Better liver, gut, and spleen histology	Higher *S. agalactiae* survival	One blend study; challenge-unit concern

Note: The table is intended to support intervention-centered tracking across the seven predefined outcome domains. “NR” indicates that no extractable outcome was available in that domain and does not demonstrate the absence of a biological effect. Cross-domain breadth represents biological or translational coherence and was not used independently to increase evidence confidence. Outcomes affected by experimental-unit concerns were interpreted as supportive rather than confirmatory evidence. FE, feed efficiency; SGR, specific growth rate; PER, protein efficiency ratio; HSP, heat-shock protein; SOD, superoxide dismutase; CAT, catalase; GPx, glutathione peroxidase; MDA, malondialdehyde; NBT, nitroblue tetrazolium; Ig, immunoglobulin; MPO, myeloperoxidase.

**Table 5:** Evidence gaps and methodological priorities for future climate-resilient functional-aquafeed research.

Evidence Gap	Current Pattern in Extracted Evidence	Why It Matters for Evidence Interpretation	Recommended Standard for Future Studies
Species imbalance	Nile tilapia is strongly represented; fewer studies on marine carnivores, salmonids, flounder, yellowtail, grouper, sea bass and sea bream.	Limits generalization across thermal guilds, feeding habits and production systems.	Include diverse finfish taxa and analyze responses by feeding habit, thermal preference and culture environment.
Thermal-protocol heterogeneity	Control and stress temperatures, exposure duration, shift rate, and recovery are inconsistently reported.	Weakens comparability and limits meta-analysis.	Report optimal temperature, stress temperature, rate of change, exposure duration, recovery period and sampling time in all studies.
Limited dose–response evidence	Some studies include dietary levels, but many evaluate a single supplemented diet.	Prevents identification of effective, marginal and excessive supplementation ranges.	Use a control and at least three doses, reporting active-compound concentrations.
Over-reliance on selected biomarkers	Most studies assess growth, redox, and immunity; few integrate gut, metabolism, and omics.	Thermal resilience is a multisystem phenotype.	Use integrated panels covering growth, stress, redox, immunity, gut, metabolism, and key genes.
Limited omics integration	Transcriptomics, metabolomics and microbiome analyses are under-represented.	Mechanistic pathways remain incomplete.	Combine multiomics with conventional physiological markers and performance outcomes.
Few recovery and disease-challenge studies	Post-stress recovery and pathogen challenge after thermal exposure are rare.	Farm relevance depends on recovery and secondary disease resistance, not only acute survival.	Include recovery sampling and pathogen challenge where ethically and biologically appropriate.
Risk-of-bias reporting gaps	Randomization, blinding and experimental-unit definition are frequently unclear.	May reduce confidence in synthesized evidence.	Use tanks as experimental units, with randomization, blinding, and complete outcome reporting.

Note: This table synthesizes major methodological limitations and research priorities identified from the current evidence map.

**Table 6:** Translational framework for climate-resilient functional-aquafeed application.

Application Phase	Nutritional Objective	Candidate Strategies	Expected Markers	Formulation Caution
Pre-stress conditioning	Improve readiness before thermal stress.	Antioxidants, phytobiotics, probiotics, organic acids, algal bioactives, amino acids.	Higher antioxidant capacity, stable immunity, improved gut condition, lower stress responses.	Avoid overstimulation; optimize dose and feeding duration.
During thermal stress	Maintain intake, digestion, redox balance, and immunity.	Digestible diets, butyrate, probiotics, antioxidant micronutrients.	Stable FE/FCR, lower MDA and stress markers, maintained immunity.	Ensure palatability and digestibility.
Post-stress recovery	Restore tissues, gut, immunity, and metabolism.	Probiotics, organic acids, vitamins, selenium, phytobiotics, algal bioactives.	Improved survival, gut recovery, normalized metabolites, better disease resistance.	Assess recovery beyond immediate endpoints.
Multi-stressor periods	Buffer thermal, water-quality, and pathogen stress.	Combined antioxidant–immune–gut formulations and probiotic–algal blends.	Higher survival, lower oxidative and tissue damage, stronger innate immunity.	Use factorial designs to test additive and synergistic effects.

Note: This framework synthesizes the practical implications of the evidence map. It should be interpreted as a research-guidance framework rather than as universal formulation recommendations.

## Data Availability

The original contributions presented in this study are included in the article and Supplementary Materials. Further inquiries can be directed to the corresponding author.

## Supplementary Materials

The following supporting information can be downloaded at https://www.mdpi.com/article/10.3390/vetsci13080756/s1. Supplementary Table S1, expanded core search strategy and source-level record counts for the PRISMA-guided evidence map; Supplementary Table S2, full-text exclusion categories and operational decision rules for PRISMA screening; Supplementary Table S3, extraction matrix for representative primary studies highlighted in the main synthesis tables; Supplementary Table S3A, DOI-verified experimental-design and basal-diet characteristics of all 28 included studies; Supplementary Table S3B, quantitative thermal-protocol characteristics and operational classifications; Supplementary Table S4A, study-level SYRCLE risk-of-bias judgments for all 28 included studies; Supplementary Table S4B, study-specific explanations supporting the SYRCLE judgments; Supplementary Table S4C, outcome-level consequences of experimental-unit concerns in the 14 affected studies; Supplementary Table S5, domain-level distribution of low-, unclear- and high-risk judgments; Supplementary Table S6A,B, study-level and quantitative aquaculture-specific reporting assessments; Supplementary File S1, complete 28-study evidence map and accompanying design, thermal-protocol and risk-of-bias worksheets.
