# Supplementary material for: Functional Aquafeeds for Climate-Resilient Finfish Culture: A PRISMA-Guided Systematic Evidence Map of Nutritional Strategies for Thermal-Stress Tolerance, Immunity and Metabolic Homeostasis
Source: Vet Sci. 2026 Jul 30;13(8):756. doi: 10.3390/vetsci13080756 (PMC13517477; doi:10.3390/vetsci13080756)
Supplement: Supplementary file 1 [file vetsci-13-00756-s001.zip › Supplementary_Tables_S1-S6.pdf]

Supplementary Tables S1–S6

Functional aquafeeds for climate-resilient finfish culture

Supplementary Table S1. Expanded core search strategy and source-level record counts for the PRISMA-guided evidence map.

Database/source	Search field	Core search string or strategy	Purpose	Records identified (n)
PubMed/MEDLINE	Title/Abstract	(fish OR finfish OR aquaculture OR cultured fish) AND (thermal stress OR heat stress OR cold stress OR temperature challenge OR high temperature OR low temperature) AND (dietary OR feed OR aquafeed OR supplementation OR functional feed)	Identify PubMed-indexed primary feeding studies linking functional feeds with thermal-stress outcomes.	94
Web of Science/Scopus	Title/Abstract/Keywords	Core thermal-stress and functional-feed search adapted to database syntax; ingredient-specific searches added for probiotics, phytogenics, vitamins, selenium, amino acids and algal bioactives.	Expand coverage beyond biomedical indexing and capture aquaculture nutrition journals.	138
Publisher databases	Article title/abstract/keywords	ScienceDirect, Wiley Online Library and SpringerLink searches using thermal-stress, aquafeed and ingredient-specific terms.	Capture articles indexed inconsistently across PubMed, Scopus or Web of Science.	92
Google Scholar	Title/abstract screening of first 200 relevance-ranked results	Focused combinations of fish, feed additive, heat stress, cold stress, antioxidant, immunity and metabolism.	Supplement database searches and identify potentially missed recent studies.	37
Citation chasing	Reference lists and cited-by searching	Backward and forward checking of included studies and relevant reviews.	Identify eligible studies missed by database syntax or indexing limitations.	25
Total	-	-	Total candidate records before duplicate removal.	386

Note. This table provides the expanded search strategy and source-level record counts used for the PRISMA-guided evidence map. Counts reflect the search log used in this review. All database and publisher-platform searches covered each source from inception or its available coverage through 5 July 2026, without a lower publication-year restriction; the Google Scholar and citation searches were completed or updated on the same date.

Supplementary Table S2. Full-text exclusion categories for the expanded PRISMA screening.

Exclusion category	Operational definition	Example decision rule	Full-text exclusions (n)
No thermal-stress component	The study evaluated functional feed under normal temperature only.	Exclude unless the stated objective directly involved thermal-tolerance mechanisms.	12
No dietary intervention	The intervention was injection, immersion, bath treatment or waterborne exposure.	Exclude because the review focuses on aquafeed-based nutritional strategies.	8
No functional dietary manipulation	The nutrient or compound was present only as a routine component of the basal diet, requirement-level formulation or vitamin–mineral premix, or was provided solely to correct a documented nutritional deficiency	Exclude unless the nutrient was deliberately manipulated as a supplemental, graded or strategically formulated dietary treatment, compared with an appropriate basal, non-supplemented or lower-dose comparator, and evaluated specifically for effects beyond routine nutrient provision under an eligible thermal-stress model. Essential and non-essential nutrients were judged using the same rule.	0
Non-finfish or non-aquaculture model	The study used an aquatic invertebrate, laboratory model fish, ornamental fish without food-aquaculture relevance, cell-culture model, isolated tissue, or ex vivo preparation.	Exclude because the review was restricted to live cultured finfish evaluated in controlled dietary feeding trials.	6
No appropriate comparator	No basal, non-supplemented, lower-dose or stress-control diet was included.	Exclude from synthesis of intervention effect.	5
Not original feeding trial	Review, conference abstract, editorial, patent, thesis or non-experimental article.	Exclude from the primary-study dataset.	7

Exclusion category	Operational definition	Example decision rule	Full-text exclusions (n)
Insufficient outcome reporting	No extractable outcome related to growth, stress, antioxidant defense, immunity, metabolism, gut health or survival.	Exclude from main synthesis but consider for background if relevant.	5
Total full-text exclusions	All excluded full-text articles after eligibility assessment.	Records not retained in the final qualitative evidence map.	43

Note. This table defines the exclusion categories used to classify full-text records during PRISMA screening.

Supplementary Table S3A. Extraction matrix for representative primary studies highlighted in the main synthesis tables.

Study	Species	Functional intervention	Thermal model	Dose/level	Feeding duration	Main outcome domains	Direction of response
Dawood et al. (2020)	Nile tilapia	Sodium butyrate	Heat stress after feeding period	0-2 g/kg diet	8 weeks	Growth, FE/FCR, survival, cortisol, glucose, HSP70, antioxidant enzymes, immunity, intestinal morphology	Positive/protective
Naiel et al. (2021)	Nile tilapia	Organic selenium	Sub-optimal temperature	0-0.6 mg/kg diet	56 days	Growth, serum biochemistry, immunity, antioxidative capacity, stress-related genes	Positive/protective
Kumar et al. (2020)	Striped catfish	Selenium nanoparticles + riboflavin	Elevated temperature + arsenic stress	NR	90 days	Growth, survival, cortisol/glucose, HSP70, lipid peroxidation, antioxidant and immune indices	Positive/protective
Ming et al. (2012)	Wuchang bream	Emodin + vitamin C	Rapid acute heat challenge after feeding	Emodin 60 mg/kg; vitamin C 700 mg/kg supplemental (approximately 750 mg/kg total dietary vitamin C); combination included both	60 days	Growth, biochemical parameters, HSP70 mRNA expression	Mixed/mechanistic
Cheng et al. (2018)	Pufferfish	Astaxanthin	High-temperature stress	80-320 mg/kg diet	56 days	Growth, ROS, SOD, CAT, HSP70, non-specific immunity	Positive/protective
Elbahnaswy et al. (2024)	Nile tilapia	*Bacillus* spp.	Heat shock	0.5 and 1% diet	58 days	Stress biomarkers, antioxidant status, immunity, histopathology, HSP genes	Positive/protective
Alrashada et al. (2023)	Nile tilapia	Propolis	Cold-stress challenge	10 g/kg reported as most effective	28 days	Growth, redox status, immune response	Positive/protective
Shehata et al. (2025)	Nile tilapia	Bay laurel essential oil	Cold stress	1.5 g/kg reported as most effective	84 days	Growth, immunity, antioxidant defense, inflammation, IGF-1, GLUT4	Positive/protective
Mahfuj et al. (2026)	Rohu	Dietary *Spirulina platensis*	Heat stress	50 g/kg diet	42 days	Growth, antioxidant response and immunity	Positive/protective
Ahmed et al. (2026)	Nile tilapia	Spirulina-CoQ10 nanoemulsion	Heat stress + Streptococcus agalactiae challenge	0, 10, 20 and 40 mg/kg diet	60 days	Growth, antioxidant, immune biomarkers, histopathology, post-challenge survival	Positive/protective

Note. NR, not reported in the source article or not extractable for the specific evidence-map field after full-text assessment. The complete 28-study evidence map is provided as Supplementary File 1 (S3A).

Supplementary Table S3B. Quantitative characteristics and operational classification of thermal-stress protocols in the 28 included studies.

ID	Study	Species	Reference/control temperature (°C)	Reference basis	Stress temperature(s) (°C)	Thermal deviation, ΔT (°C)	Temperature-change pattern/rate	Thermal-exposure duration	Recovery/post-stress phase	Additional stressor	Operational duration class	Final operational thermal-model classification
1	Ming et al. (2012)	Wuchang bream	25	Concurrent challenge-start/control temperature	34	+9	Direct transfer from 25 to 34°C	Biomarker sampling at 0, 2, 6, and 12 h; mortality followed to 24 h	None reported	None	Rapid acute	Rapid acute heat challenge (direct transfer)
2	Cheng et al. (2018a)	Pufferfish	NR	Not extractable from the accessible final report	NR	NR	Fixed high-temperature challenge after an 8-week feeding period; exact temperature and ramp rate NR	48 h	None reported	None	Short-term	Short-term post-feeding heat challenge
3	Cheng et al. (2018b)	Pufferfish	25	Concurrent control and challenge-start temperature	25, 21, 17, and 13	−12 to final set point	Progressive cooling from 25 to 13°C at approximately 1°C/h; samples collected after 1 h at each stated temperature	Completed within 24 h; approximately 12 h to reach 13°C, with 1-h sampling exposure at stated temperatures	None reported	None	Rapid acute	Rapid acute progressive cold challenge
4	Dawood et al. (2020a)	Nile tilapia	24.1 ± 0.3	Concurrent rearing temperature before the challenge	32	Approximately +7.9	Transferred to and maintained at 32°C; ramp rate NR	Up to 48 h; mortality monitored during the challenge	None reported	None	Short-term	Short-term fixed heat challenge
5	Kumar et al. (2020)	Striped catfish	NR	Concurrent control temperature not extracted	34	NR	Fixed elevated temperature maintained during the feeding experiment	90 d	None reported	Arsenic exposure	Prolonged	Prolonged fixed high-temperature plus arsenic exposure
6	Naiel et al. (2021)	Nile tilapia	26–28	Source-reported species optimum range; not a concurrent control	21.50 ± 0.50	−4.5 to −6.5 versus reported optimum range	Fixed suboptimal temperature throughout feeding	56 d	None reported	None	Prolonged	Prolonged fixed suboptimal-cold rearing
7	Abdel-Ghany et al. (2021)	Nile tilapia	25 ± 1	Concurrent feeding/rearing temperature before the tolerance test	Progressive cooling; mortality reported from 16 to 6	Approximately −9 to −19 across reported mortality temperatures	Progressive cooling to a lethal-temperature endpoint; exact rate NR	Endpoint-dependent; no fixed exposure duration reported	None reported	None	Endpoint-defined	Endpoint-defined progressive cold-to-lethal-endpoint challenge
8	Abdel-Ghany et al. (2019)	Nile tilapia	25	Concurrent feeding/rearing temperature before the tolerance test	16, 15, 12, 10, 9, 8, 7, and 6	−9 to −19	Progressive cooling with mortality recorded at successive temperatures; exact rate NR	Endpoint-dependent; no fixed exposure duration reported	None reported	None	Endpoint-defined	Endpoint-defined progressive cold-to-lethal-endpoint challenge
9	Yousefi et al. (2022)	Rainbow trout	16	Thermal-challenge starting temperature	22	+6	Gradual warming from 16 to 22°C over 7 d	7 d	None reported	None	Short-term	Short-term gradual warming challenge
10	Abdel-Ghany et al. (2023)	Nile tilapia	25	Concurrent challenge-start temperature	40	+15	Temperature increased from 25 to 40°C over 3 h, then held at 40°C for 4 h (approximately 5°C/h during ramp)	7 h total	None reported	None	Rapid acute	Rapid acute heat ramp-and-hold challenge
11	Alrashada et al. (2023)	Nile tilapia	26	Concurrent normal-temperature factorial control	18	−8	Fixed temperatures maintained during the feeding period	28 d	None reported	None	Prolonged	Prolonged fixed cold rearing (factorial design)
12	Elbahnaswy et al. (2024)	Nile tilapia	NR	Pre-challenge reference temperature not extracted	36 ± 0.5	NR	Acute heat shock; ramp rate NR	2 h	None reported	None	Rapid acute	Rapid acute heat-shock challenge
13	Akram et al. (2024)	Asian seabass	28	Concurrent acclimation and recovery temperature	CTmin approximately 13.20–14.03	Approximately −14.80 to −13.97	Progressive cooling at 0.21 ± 0.04°C/min until loss of equilibrium/CTmin endpoint	Endpoint-dependent	Returned to 28°C and observed for 24 h	None	Endpoint-defined	Endpoint-defined CTmin progressive-cooling challenge

ID	Study	Species	Reference/control temperature (°C)	Reference basis	Stress temperature(s) (°C)	Thermal deviation, ΔT (°C)	Temperature-change pattern/rate	Thermal-exposure duration	Recovery/post-stress phase	Additional stressor	Operational duration class	Final operational thermal-model classification
14	Islam et al. (2024)	European seabass	NR	Concurrent pre-heatwave temperature not extracted	32	NR	Fixed 32°C heatwave after the feeding phase; transition rate NR	18 d	None reported	None	Prolonged	Prolonged fixed heatwave exposure
15	Behairy et al. (2024)	Nile tilapia	25	Concurrent normal-temperature control	18	−7	Fixed cold temperature maintained during the feeding experiment	60 d	None reported	None	Prolonged	Prolonged fixed cold exposure
16	Mohapatra et al. (2014)	Rohu	28	Lowest-temperature factorial control and reported ideal range upper value	28, 31, 34, and 37	0, +3, +6, and +9	Fixed factorial temperature levels	30 d	None reported	None	Prolonged	Prolonged factorial fixed-temperature rearing
17	Youssuf et al. (2025)	Nile tilapia	28 ± 2	Concurrent feeding/rearing temperature before challenge	12	Approximately −16	Direct transfer to pre-cooled, aerated 12°C water	30 min	None reported	Separate hypoxia challenge (DO 1.5–3.0 mg/L)	Rapid acute	Rapid acute cold challenge; separate hypoxia challenge
18	Albaqami and Monier (2025)	Nile tilapia	NR	Reference temperature not sufficiently reported for extraction	36	NR	Post-feeding heat exposure; transition rate NR	NR	None reported	Separate salinity-stress arm (28 g/L)	NR/unclassified	NR/unclassified post-feeding heat challenge; separate salinity arm
19	Abu-Zahra et al. (2025)	Nile tilapia	25	Concurrent mid-temperature control	17 and 33	−8 and +8	Temperature adjusted from 25°C by 2°C/day over 4 d, then maintained at 17 or 33°C	30 d at the assigned thermal condition	None reported	None	Prolonged	Prolonged bidirectional fixed-temperature rearing after gradual adjustment
20	Shehata et al. (2025)	Nile tilapia	NR	Normal-temperature comparator not extracted	16	NR	Fixed cold temperature throughout feeding	84 d	None reported	None	Prolonged	Prolonged fixed cold rearing at 16°C
21	Yousefi et al. (2025)	Rainbow trout	17	Pre-challenge rearing/recovery temperature	25	+8	Transferred from 17 to 25°C; ramp rate NR	96 h	Returned to/recovery assessed at 17°C	None	Short-term	Short-term post-feeding heat challenge
22	Zhao et al. (2025)	Largemouth bass	NR	Pre-challenge reference temperature not extracted	33.00 ± 0.16	NR	Fixed high-temperature challenge after 56 d feeding; transition rate NR	7 d	None reported	None	Short-term	Short-term post-feeding heat challenge
23	Zhang et al. (2025)	Hybrid crucian carp HCC2	26	Concurrent challenge-start temperature	38	+12	Temperature increased from 26 to 38°C over 6 h (approximately 2°C/h), then maintained to complete 24 h	24 h total	None reported	None	Rapid acute	Rapid acute heat ramp-and-hold challenge
24	Li et al. (2023)	Rainbow trout	18	Concurrent challenge-start temperature	24	+6	Temperature increased from 18 to 24°C within 30 min, then held at 24°C	8.5 h total (0.5-h ramp + 8-h hold)	None reported	None	Rapid acute	Rapid acute heat ramp-and-hold challenge
25	Kumar et al. (2024)	Striped catfish	NR	Concurrent control temperature not extracted	34	NR	Fixed high temperature maintained throughout the experiment	105 d	None reported	Arsenic; low pH 6.5 in the combined-stressor arm	Prolonged	Prolonged fixed high-temperature multi-stressor exposure
26	Dawood et al. (2020b)	Nile tilapia	25–28	Source-reported optimal/rearing range; concurrent challenge reference not verified	32	+4 to +7 versus reported range	Post-feeding fixed 32°C heat-resistance test; transition rate NR	Up to 48 h; test terminated at 50% mortality	None reported	None	Short-term	Short-term endpoint-terminated heat-resistance test
27	Mahfuj et al. (2026)	Rohu	30	Concurrent ambient-temperature control	36	+6	Fixed ambient and heat-stress temperatures maintained concurrently	42 d	None reported	Water-administered probiotic arm; dietary Spirulina arm	Prolonged	Prolonged fixed high-temperature rearing
28	Ahmed et al. (2026)	Nile tilapia	25 ± 0.5	Concurrent normal-temperature control	32 ± 0.5	+7	Fixed temperatures maintained throughout feeding	60 d	Post-thermal Streptococcus agalactiae challenge	Subsequent bacterial challenge	Prolonged	Prolonged fixed high-temperature rearing plus post-thermal pathogen challenge

Note. ΔT = Tstress − Treference. Positive values indicate heat exposure and negative values indicate cold exposure. Rapid acute, ≤24 h; short-term, >24 h to 7 days; prolonged, >7 days; endpoint-defined, progressive warming or cooling until a physiological, behavioral or mortality endpoint; NR/unclassified, insufficient protocol information. Concurrent control, acclimation or challenge-start temperatures were prioritized as reference conditions; species-optimal ranges were used only descriptively when reported in the source study. Additional environmental, chemical or biological stressors were retained as modifiers rather than separate duration categories. These are review-specific evidence-mapping definitions and not universal species-independent physiological thresholds. Full DOI-linked verification notes and source URLs are provided in Supplementary File 1.

Supplementary Table S4A. Study-level SYRCLE risk-of-bias judgments for all 28 included studies.

ID	Study	SG	BS	AC	RH	CIB	ROA	OAB	IOD	SOR	OB
1	Ming et al. (2012)	U	L	U	L	U	U	U	U	U	H
2	Cheng et al. (2018a)	U	L	U	U	U	U	U	U	U	U
3	Cheng et al. (2018b)	U	L	U	U	U	U	U	U	U	U
4	Dawood et al. (2020a)	U	L	U	L	U	L	U	L	U	L
5	Kumar et al. (2020)	U	L	U	L	U	U	U	L	U	H
6	Naiel et al. (2021)	U	L	U	U	U	U	U	L	U	U
7	Abdel-Ghany et al. (2021)	U	L	U	U	U	U	U	U	U	U
8	Abdel-Ghany et al. (2019)	U	L	U	U	U	U	U	U	U	H
9	Yousefi et al. (2022)	U	L	U	L	U	U	U	L	U	H
10	Abdel-Ghany et al. (2023)	U	L	U	U	U	U	U	L	U	H
11	Alrashada et al. (2023)	U	L	U	L	U	U	U	L	U	H
12	Elbahnaswy et al. (2024)	U	L	U	U	U	L	U	L	U	H
13	Akram et al. (2024)	U	L	U	L	U	L	U	L	U	H
14	Islam et al. (2024)	U	L	U	L	U	U	U	L	U	H
15	Behairy et al. (2024)	U	L	U	L	U	L	U	L	U	H
16	Mohapatra et al. (2014)	U	L	U	L	U	U	U	L	U	L
17	Youssuf et al. (2025)	U	L	U	L	U	L	U	U	U	U
18	Albaqami and Monier (2025)	U	L	U	L	U	U	U	L	U	U
19	Abu-Zahra et al. (2025)	U	L	U	L	U	U	U	L	U	H
20	Shehata et al. (2025)	U	L	U	U	U	U	U	L	U	U
21	Yousefi et al. (2025)	U	L	U	U	U	U	U	L	U	U
22	Zhao et al. (2025)	U	L	U	L	U	U	U	L	U	U
23	Zhang et al. (2025)	U	L	U	L	U	L	U	L	U	H
24	Li et al. (2023)	U	L	U	U	U	U	U	U	U	U
25	Kumar et al. (2024)	U	L	U	U	U	U	U	L	U	H
26	Dawood et al. (2020b)	U	L	U	U	U	U	U	L	U	U
27	Mahfuj et al. (2026)	U	L	U	U	U	U	U	U	U	U
28	Ahmed et al. (2026)	U	L	U	L	U	U	U	L	U	H

Note. L, low risk; U, unclear risk; H, high risk. SG, sequence generation; BS, baseline similarity; AC, allocation concealment; RH, random housing/tank allocation; CIB, caregiver/investigator blinding; ROA, random outcome assessment; OAB, outcome-assessor blinding; IOD, incomplete outcome data; SOR, selective outcome reporting; OB, other bias. An unclear judgment indicates insufficient reporting and does not by itself demonstrate poor methodological conduct.

Supplementary Table S4B. Study-specific explanations supporting the SYRCLE judgments.

ID	Study	Key study-specific justification
1	Ming et al. (2012)	Random treatment allocation and triplicate tanks were reported, but the sequence method, concealment and blinding were not. Multiple fish sampled per tank were analysed with n=9 rather than tank as the replicate, creating an explicit unit-of-analysis concern.

ID	Study	Key study-specific justification
2	Cheng et al. (2018a)	Comparable starting fish and replicated dietary groups were reported. The randomization method, concealment, blinding, random sampling procedure, attrition flow and tank-level analytical unit were insufficiently described in the published report.
3	Cheng et al. (2018b)	Comparable starting fish and replicated dietary groups were reported. Allocation sequence, concealment, blinding, random outcome selection, attrition flow and the analytical treatment of tank subsamples were insufficiently described.
4	Dawood et al. (2020a)	Fish were randomly allocated to 15 aquaria with triplicate tanks. The heat challenge retained replication, and one liver sample per aquarium was randomly selected; reported analyses were consistent with tank-level replication.
5	Kumar et al. (2020)	A completely randomized block design with triplicate tanks was reported. However, the authors explicitly treated individual fish as experimental units for biochemical and immune outcomes after finding no tank effect, despite diet being delivered at tank level.
6	Naiel et al. (2021)	Comparable baseline groups and replicated diets were reported. The random sequence method, concealment, blinding, random sampling and analytical handling of within-tank fish were not described sufficiently for a low-risk judgment.
7	Abdel-Ghany et al. (2021)	Replicated dietary lipid treatments and comparable starting fish were reported. Random allocation, concealment, blinding, attrition flow and whether tank or individual fish was the analytical unit were not adequately documented.
8	Abdel-Ghany et al. (2019)	Triplicate aquaria and comparable initial fish were reported, but random allocation was not. Only a small number of fish per treatment were sampled for several endpoints, and analyses did not account clearly for tank, indicating a unit-of-analysis concern.
9	Yousefi et al. (2022)	Fish were randomly distributed among 12 tanks with three tanks per diet. Multiple fish per tank were sampled and analysed as n=6 without a tank-level term, indicating likely pseudoreplication for physiological endpoints.
10	Abdel-Ghany et al. (2023)	Twenty-one aquaria and three replicates per treatment were reported, but initial randomization was not clearly described. Blood and tissue outcomes used fish-level sample sizes without an explicit tank-level model, creating a unit-of-analysis concern.
11	Alrashada et al. (2023)	A replicated factorial aquarium design and comparable baselines were reported. Three fish per replicate were used for blood outcomes and analysed as individual observations, producing a unit-of-analysis concern.
12	Elbahnaswy et al. (2024)	Triplicate tanks were reported and fish for post-feeding sampling were randomly selected. Initial allocation and blinding were unclear; biochemical and gene-expression results were analysed with fish-level n values rather than tank-level replication.
13	Akram et al. (2024)	Fish were randomly placed in duplicate tanks and outcome fish were randomly selected. Biological outcomes used fish-level n values without incorporating tank as the experimental unit, creating a unit-of-analysis concern.
14	Islam et al. (2024)	Fish and diets were randomly allocated to three replicate tanks per treatment. Serum pooling supported tank-level replication, but gene-expression results used n=6 with only three tanks and no hierarchical tank term, creating a mixed unit-of-analysis concern.
15	Behairy et al. (2024)	Fish were arbitrarily allocated to four replicate groups and some outcome sampling was random. Physiological and molecular outcomes used 12 fish per treatment as independent observations rather than four tank replicates.
16	Mohapatra et al. (2014)	Fish were randomly distributed in a completely randomized factorial design with three replicates. Fish-level apoptosis data were averaged within replicate tanks and analysed using tank means, supporting an appropriate experimental unit.
17	Youssuf et al. (2025)	Fish were randomized into triplicate dietary groups and outcome fish were randomly selected. The combined cold/hypoxia challenge and sample-flow reporting were not fully consistent, so attrition and analytical-unit judgments remained unclear.
18	Albaqami and Monier (2025)	A total of 270 fish were impartially allocated to 18 aquaria with three replicates per diet and comparable baselines. Three fish per aquarium were sampled, but the published report did not make the tank-level aggregation/model sufficiently clear.
19	Abu-Zahra et al. (2025)	Fish were haphazardly distributed among 18 aquaria with triplicate groups. Three fish per aquarium were analysed as n=9 per treatment in two-way analyses, indicating a unit-of-analysis concern.
20	Shehata et al. (2025)	Replicated dose groups and comparable starting fish were reported. Random allocation sequence, concealment, blinding, random outcome selection and treatment of within-tank subsamples were not sufficiently described.
21	Yousefi et al. (2025)	Triplicate dietary groups (240 fish in 12 tanks) and comparable starting fish were reported. The initial allocation, random sampling and tank-level analytical treatment of physiological samples were insufficiently described.
22	Zhao et al. (2025)	A total of 360 uniform fish were randomly selected and distributed to 18 buckets, with triplicate tanks per diet. Sampling and the statistical unit for post-stress biochemical and molecular outcomes were not described clearly enough for a low-risk unit judgment.
23	Zhang et al. (2025)	Fish were randomly divided into five dietary groups with three replicate cages and outcome fish were randomly selected. For heat challenge, fish from each treatment were combined into a single challenge tank, collapsing treatment replication.
24	Li et al. (2023)	Comparable full-sib juveniles and dietary groups were described, but the accessible report did not provide enough detail on allocation, blinding, attrition, random sampling or tank-level statistical analysis.
25	Kumar et al. (2024)	Triplicate tanks were reported but allocation procedures were not clearly randomized. Multiple fish per replicate were sampled for serum and molecular outcomes and analysed without an explicit tank-level model, indicating a unit-of-analysis concern.

ID	Study	Key study-specific justification
26	Dawood et al. (2020b)	Replicated dietary groups and comparable starting fish were reported. Randomization, blinding, random sampling and the tank-level analytical unit for physiological outcomes were not sufficiently described in the published report.
27	Mahfuj et al. (2026)	Four treatment groups were reported: an ambient-temperature control at 30 °C, a heat-stress control at 36 °C, a water-administered probiotic group at 36 °C and a dietary *Spirulina platensis* group receiving 50 g/kg diet at 36 °C for 42 days. Only the dietary *Spirulina* arm was included in the aquafeed synthesis. However, tank replication, random allocation, blinding, attrition and analytical-unit handling were not reported in sufficient detail.
28	Ahmed et al. (2026)	A completely randomized design with five diets and three replicate tanks was reported. Histology and challenge outcomes used multiple fish per treatment in one-way analyses without an explicit tank-level term, indicating a unit-of-analysis concern.

Note. Judgments were based on the information reported in the final peer-reviewed article. Full source URLs and the coded assessment are provided in Supplementary File 1.

Supplementary Table S4C. Outcome-level consequences of experimental-unit concerns in 14 included studies.

Evidence-map ID	Study	Experimental-unit or replication concern	Outcomes principally affected	Consequence for evidence interpretation
1	Ming et al. (2012)	Multiple fish sampled from replicate tanks were analysed as n = 9 rather than using tank as the replicate.	Biochemical parameters, HSP70 mRNA expression and other sampled fish-level endpoints.	Mechanistic findings were treated as hypothesis-generating. Tank-level growth evidence was retained only where based on independent replicate-tank means.
5	Kumar et al. (2020)	Individual fish were explicitly treated as experimental units for biochemical and immune analyses although the diet was assigned at tank level.	Cortisol/glucose, HSP70, lipid peroxidation, antioxidant indices and immune indices.	Physiological and immune effects were assigned reduced confidence because within-tank clustering may have been ignored; tank-based performance outcomes were not automatically downgraded.
8	Abdel-Ghany et al. (2019)	Fish sampled from triplicate aquaria were analysed without clear adjustment for the tank-level dietary allocation.	Fatty-acid composition, cold-tolerance and other sampled physiological endpoints.	Response direction was retained, but the precision of fish-level comparisons was considered uncertain. Tank-level growth and feed-utilization results were interpreted separately.
9	Yousefi et al. (2022)	Multiple fish per tank were analysed as n = 6 without a tank term in the statistical model.	Physiological responses, antioxidant status, hepatic-damage markers and stress responses.	Fish-level mechanistic outcomes were considered supportive rather than confirmatory because variance may have been underestimated.
10	Abdel-Ghany et al. (2023)	Blood and tissue outcomes used fish-level sample sizes without an explicit tank-level or hierarchical model.	Hematology, cortisol, glucose, IgM, complement, serum biochemistry and tissue-related heat-stress responses.	Mechanistic evidence was interpreted cautiously; statistical significance was not treated as equivalent to evidence based on independent tank replicates.
11	Alrashada et al. (2023)	Three fish from each replicate were analysed as individual observations for blood outcomes.	Blood biochemical, redox and immune-response endpoints.	Growth evidence was retained where tank based, whereas blood, redox and immune findings were downgraded to supportive evidence.
12	Elbahnaswy et al. (2024)	Biochemical and gene-expression data used fish-level n values rather than tank-level replication.	Stress biomarkers, antioxidant and immune measurements, and HSP-related gene expression.	Biomarker and gene-expression findings were interpreted as supportive and were not used alone to establish strong dietary efficacy.
13	Akram et al. (2024)	Biological outcomes used fish-level observations without incorporating tank as the experimental unit.	IGF-1, antioxidant-gene expression, physiological responses and cold-tolerance endpoints.	Fish-level physiological and molecular effects were assigned reduced confidence; tank-based growth and FCR outcomes were considered separately where applicable.
14	Islam et al. (2024)	Serum was pooled at tank level, but gene-expression results used n = 6 from only three tanks without a hierarchical tank term.	HSP70, GLUT2, FADS2 and other gene-expression outcomes.	Tank-pooled serum results were considered more reliable than the gene-expression evidence, which was treated as supportive because of the mixed analytical-unit approach.
15	Behairy et al. (2024)	Twelve fish per treatment were analysed as independent observations rather than four replicate tanks.	Physiological, immune-antioxidant, behavioural, histological and molecular outcomes.	Fish-level mechanistic claims were downgraded because the effective replication may have been overstated; tank-based production outcomes were evaluated separately.
19	Abu-Zahra et al. (2025)	Three fish per aquarium were analysed as n = 9 per treatment in two-way analyses.	Physiological, immunological, behavioural and stress-response endpoints.	The response direction was retained, but confidence in p-values and precision was reduced because fish from the same aquarium were not demonstrably modelled as clustered observations.
23	Zhang et al. (2025)	Fish from replicate cages within each dietary treatment were combined into one heat-challenge tank, collapsing independent treatment replication.	All heat-challenge-specific survival, stress, HSP70, antioxidant and metabolomic outcomes.	Challenge-specific findings were treated as unreplicated treatment-level observations. Pre-challenge outcomes were not automatically affected when based on independent cages.
25	Kumar et al. (2024)	Multiple fish per replicate were analysed for serum and molecular outcomes without an explicit tank-level model.	Serum biochemistry, antioxidant/HSP/immune genes and DNA-damage endpoints.	Serum and molecular findings were considered supportive rather than confirmatory; independently replicated tank-level growth outcomes were interpreted separately.
28	Ahmed et al. (2026)	Histology and challenge outcomes used multiple fish per treatment in one-way analyses without an explicit tank-level term.	Histopathology and pathogen-challenge outcomes, including post-challenge survival.	Histological and challenge conclusions were interpreted cautiously because the analytical model did not clearly preserve tank-level independence.

Note. The 14 studies listed here correspond to a high-risk judgment in the SYRCLE ‘other bias’ domain and a ‘Concern’ judgment for tank replication/experimental unit in Supplementary Table S6. Among these 14 studies, 13 involved fish-level observations or within-tank subsamples analysed without adequately accounting for tank-level clustering, whereas one study combined replicate cages into a single treatment-level tank during the thermal challenge and thereby removed independent challenge replication. Such analytical approaches may underestimate sampling variance, narrow confidence intervals and increase the likelihood of apparently significant treatment effects. The affected-outcome assessment was therefore applied at the outcome level and does not imply that every result within a study was invalid. Tank-based growth or feed-utilization outcomes were not automatically downgraded when they were analysed using independent replicate-tank means. Fish-level biochemical, immune, histological and molecular outcomes that did not account for tank clustering were treated as supportive or hypothesis-generating evidence, and challenge-specific findings from the collapsed-replication design were treated as unreplicated treatment-level observations.

Supplementary Table S5. Domain-level distribution of SYRCLE risk-of-bias judgments across the 28 included studies.

Risk-of-bias domain	Low, n	Low, %	Unclear, n	Unclear, %	High, n	High, %
Sequence generation	0	0.0	28	100.0	0	0.0
Baseline similarity	28	100.0	0	0.0	0	0.0
Allocation concealment	0	0.0	28	100.0	0	0.0
Random housing/tank allocation	15	53.6	13	46.4	0	0.0
Caregiver/investigator blinding	0	0.0	28	100.0	0	0.0
Random outcome assessment	6	21.4	22	78.6	0	0.0
Outcome-assessor blinding	0	0.0	28	100.0	0	0.0
Incomplete outcome data	20	71.4	8	28.6	0	0.0
Selective outcome reporting	0	0.0	28	100.0	0	0.0
Other bias	2	7.1	12	42.9	14	50.0
All domain-level judgments (n = 280)	71	25.4	195	69.6	14	5.0

Note. Percentages in each domain use 28 studies as the denominator. The final row summarizes all 280 judgments generated from 28 studies × 10 domains. Percentages may not total exactly 100.0 because of rounding.

Supplementary Table S6A. Aquaculture-specific reporting assessment for all 28 included studies.

ID	Study	Tank replication/experimental unit	Diet formulation/dose clarity	Thermal-protocol clarity
1	Ming et al. (2012)	Concern	Unclear	Unclear
2	Cheng et al. (2018a)	Unclear	Adequate	Adequate
3	Cheng et al. (2018b)	Unclear	Adequate	Adequate
4	Dawood et al. (2020a)	Adequate	Adequate	Adequate
5	Kumar et al. (2020)	Concern	Unclear	Adequate
6	Naiel et al. (2021)	Unclear	Adequate	Adequate
7	Abdel-Ghany et al. (2021)	Unclear	Adequate	Adequate
8	Abdel-Ghany et al. (2019)	Concern	Adequate	Adequate
9	Yousefi et al. (2022)	Concern	Adequate	Adequate
10	Abdel-Ghany et al. (2023)	Concern	Adequate	Adequate
11	Alrashada et al. (2023)	Concern	Adequate	Adequate
12	Elbahnaswy et al. (2024)	Concern	Adequate	Adequate
13	Akram et al. (2024)	Concern	Adequate	Adequate
14	Islam et al. (2024)	Concern	Adequate	Adequate
15	Behairy et al. (2024)	Concern	Adequate	Adequate
16	Mohapatra et al. (2014)	Adequate	Adequate	Adequate
17	Youssuf et al. (2025)	Unclear	Adequate	Unclear
18	Albaqami and Monier (2025)	Unclear	Adequate	Adequate
19	Abu-Zahra et al. (2025)	Concern	Adequate	Adequate
20	Shehata et al. (2025)	Unclear	Adequate	Adequate
21	Yousefi et al. (2025)	Unclear	Adequate	Adequate
22	Zhao et al. (2025)	Unclear	Adequate	Adequate
23	Zhang et al. (2025)	Concern	Adequate	Adequate
24	Li et al. (2023)	Unclear	Adequate	Adequate
25	Kumar et al. (2024)	Concern	Adequate	Adequate
26	Dawood et al. (2020b)	Unclear	Adequate	Adequate
27	Mahfuj et al. (2026)	Unclear	Adequate	Adequate
28	Ahmed et al. (2026)	Concern	Adequate	Adequate

Note. These aquaculture-specific domains were evaluated separately from the formal SYRCLE domains. Adequate indicates sufficiently clear reporting; Unclear indicates insufficient information; Concern indicates an explicit experimental-unit or replication problem.

Supplementary Table S6B. Quantitative summary of aquaculture-specific reporting judgments.

Reporting domain	Adequate, n (%)	Unclear, n (%)	Concern, n (%)
Tank replication/experimental unit	2 (7.1%)	12 (42.9%)	14 (50.0%)
Diet formulation/dose clarity	26 (92.9%)	2 (7.1%)	0 (0.0%)
Thermal-protocol clarity	26 (92.9%)	2 (7.1%)	0 (0.0%)

Note. Percentages use 28 studies as the denominator.
