# Supplementary material for: Functional Aquafeeds for Climate-Resilient Finfish Culture: A PRISMA-Guided Systematic Evidence Map of Nutritional Strategies for Thermal-Stress Tolerance, Immunity and Metabolic Homeostasis
Source: Vet Sci. 2026 Jul 30;13(8):756. doi: 10.3390/vetsci13080756 (PMC13517477; doi:10.3390/vetsci13080756)
Supplement: Supplementary file 1 [file vetsci-13-00756-s001.zip › _PRISMA_2020_Checklist.pdf]

PRISMA 2020 Checklist

Functional aquafeeds for climate-resilient finfish culture: A PRISMA-guided systematic evidence map of nutritional strategies for thermal-stress tolerance, immunity and metabolic homeostasis

Item	Section	Topic	PRISMA 2020 reporting item	Location in manuscript/supplement	Status	Description
**1**	Title	Title	Identify the report as a systematic review.	Title page: article type “Systematic Review” and title.	**Reported**	The report is explicitly identified as a systematic review and as a PRISMA-guided systematic evidence map.
**2**	Abstract	Abstract	See the PRISMA 2020 for Abstracts checklist.	Abstract; Simple Summary.	**Reported**	Objectives, search cutoff, record counts, synthesis, main findings and limitations are reported
**3**	Introduction	Rationale	Describe the rationale for the review in the context of existing knowledge.	Introduction, especially the final rationale paragraphs.	**Reported**	The manuscript explained the fragmented evidence base, relevant mechanisms and the need for a PRISMA-guided evidence map.
**4**	Introduction	Objectives	Provide an explicit statement of the objective(s) or question(s) the review addresses.	End of Introduction; Section 2.1.	**Reported**	Four objectives and an explicit research question were stated.
**5**	Methods	Eligibility criteria	Specify the inclusion and exclusion criteria and how studies were grouped for the syntheses.	Sections 2.2 and 2.7.	**Reported**	PICOS eligibility, report restrictions and operational functional-intervention criteria were defined; synthesis groupings were described in Section 2.7.
**6**	Methods	Information sources	Specify all sources searched or consulted and the date each source was last searched or consulted.	Section 2.3; Supplementary Table S1.	**Reported**	Sources and the overall final search date (5 July 2026) were reported. several sources are grouped in Supplementary Table S1.
**7**	Methods	Search strategy	Present the full search strategies for all databases, registers and websites, including filters and limits.	Section 2.3; Supplementary Table S1.	**Reported**	A PubMed/MEDLINE core string was provided, but Web of Science and Scopus were combined and publisher-platform and Google Scholar searches were summarized rather than reproduced as complete source-specific strategies. CAB Abstracts was not searched and has been removed from this checklist.
**8**	Methods	Selection process	Specify the study-selection methods, including the number and independence of reviewers and disagreement resolution.	Section 2.4.	**Reported**	Two reviewers independently screened records and full texts; disagreements were resolved by discussion with third-reviewer consultation when required.
**9**	Methods	Data collection process	Specify the data-collection methods, including the number and independence of reviewers, investigator contact and any automation tools.	Section 2.5.	**Reported**	A standardized, pilot-tested extraction form and missing-data procedures were described. The number and independence of data extractors, the figure-extraction software and details/results of author contact were not reported.
**10a**	Methods	Data items: outcomes	List and define all outcomes sought and explain how compatible results were selected.	Sections 2.5 and 2.7.	**Reported**	Outcome domains were extensively defined.
**10b**	Methods	Data items: other variables	List and define all other variables sought and describe assumptions about missing or unclear information.	Sections 2.5 and 2.7; Supplementary Tables S3A and S3B; Supplementary File 1.	**Reported**	Study, diet, design and thermal-protocol variables were listed, and assumptions for reference temperature, thermal deviation and NR/unclassified coding were described.
**11**	Methods	Study risk-of-bias assessment	Specify the risk-of-bias methods, tool(s), reviewer process and any automation used.	Section 2.6; Supplementary Tables S4A–S4C, S5 and S6A–S6B.	**Reported**	The adapted SYRCLE tool, domains, judgment rules, aquaculture-specific assessment and use in interpretation were described.
**12**	Methods	Effect measures	Specify the effect measure(s) used for each outcome in the synthesis or presentation of results.	Sections 2.7 and 2.8.	**Reported**	No quantitative effect measure was synthesized. The prespecified qualitative comparison measure was response direction: positive, negative, neutral or mixed.

PRISMA 2020 Checklist

Item	Section	Topic	PRISMA 2020 reporting item	Location in manuscript/supplement	Status	Description
**13a**	Methods	Synthesis methods: eligibility	Describe how studies were determined to be eligible for each synthesis.	Section 2.7; Table 1; Supplementary File 1.	**Reported**	Studies were assigned to mutually exclusive intervention classes and mapped across predefined thermal-model and outcome domains.
**13b**	Methods	Synthesis methods: data preparation	Describe methods used to prepare data for presentation or synthesis.	Sections 2.5 and 2.7.	**Reported**	Figure-derived numerical extraction, missing-data handling, operational thermal classification and response-direction coding were described.
**13c**	Methods	Synthesis methods: display	Describe methods used to tabulate or visually display individual-study and synthesis results.	Section 2.7; Figures 2–4; Tables 1–6; Supplementary materials.	**Reported**	The manuscript described evidence-map matrices, hierarchical classifications, synthesis tables, thermal-model displays and the intervention-by-outcome matrix.
**13d**	Methods	Synthesis methods: approach	Describe the synthesis methods and justify the approach; report statistical methods if used.	Sections 2.7 and 2.8.	**Reported**	Narrative synthesis was guided by SWiM, and the absence of meta-analysis was justified by substantial biological and methodological heterogeneity.
**13e**	Methods	Synthesis methods: heterogeneity	Describe methods used to explore possible causes of heterogeneity.	Sections 2.7 and 2.8; Sections 3.4 and 3.13; Tables 2 and 5.	**Reported**	Variation was explored informally by species, intervention class, thermal model, dose, feeding duration, outcome domain and methodological limitations.
**13f**	Methods	Synthesis methods: sensitivity analyses	Describe any sensitivity analyses conducted.	Section 2.8.	**N/A**	No sensitivity analyses were conducted because no quantitative synthesis was performed.
**14**	Methods	Reporting-bias assessment	Describe methods used to assess risk of bias due to missing results in a synthesis.	Section 2.8; Section 4.9.	**N/A**	No formal reporting-bias or small-study-effects assessment was conducted. Potential language and publication bias were acknowledged as review limitations.
**15**	Methods	Certainty assessment	Describe methods used to assess certainty or confidence in the body of evidence.	Section 2.9.	**Reported**	Qualitative confidence factors and categories are defined, but the number and independence of assessors and an explicit decision process applied to each synthesis were not reported.
**16a**	Results	Study selection: flow	Describe the search and selection results from records identified to studies included, ideally with a flow diagram.	Section 3.1; Figure 1.	**Reported**	The manuscript reports 386 records identified, 112 duplicates removed, 274 records screened, 71 full texts assessed, 43 excluded and 28 studies included.
**16b**	Results	Study selection: excluded studies	Cite studies that might appear eligible but were excluded and explain why.	Section 3.1; Supplementary Table S2.	**Reported**	Exclusion categories, operational rules and counts were reported, but no article-level list cites potentially eligible excluded reports with a specific reason for each exclusion.
**17**	Results	Study characteristics	Cite each included study and present its characteristics.	Section 3.2; Table 1; Supplementary Tables S3, S3A and S3B; Supplementary File 1.	**Reported**	Included studies were cited and study-level species, intervention, dose, design, diet and thermal-protocol characteristics were presented in the supporting materials.
**18**	Results	Risk of bias in studies	Present risk-of-bias assessments for each included study.	Section 3.12; Supplementary Tables S4A–S4C, S5 and S6A–S6B.	**Reported**	Study-level SYRCLE judgments, supporting explanations, outcome-level experimental-unit consequences and domain-level summaries were presented.
**19**	Results	Results of individual studies	For all outcomes, present study-level summary statistics and effect estimates with precision where applicable.	Sections 3.5–3.11; Tables 1–4; Supplementary Table S3; Supplementary File 1.	**Reported**	Study-level response directions and narrative findings were presented.
**20a**	Results	Synthesis results: contributing studies	For each synthesis, summarize the characteristics and risk of bias of contributing studies.	Sections 3.3–3.13; Tables 1–5; Supplementary risk-of-bias tables.	**Reported**	Study characteristics and class-level limitations were summarized.
**20b**	Results	Synthesis results: statistical syntheses	Present the results of all statistical syntheses conducted.	Section 2.8.	**N/A**	No statistical synthesis or meta-analysis was conducted.

PRISMA 2020 Checklist

Item	Section	Topic	PRISMA 2020 reporting item	Location in manuscript/supplement	Status	Description
**20c**	Results	Synthesis results: heterogeneity	Present results of investigations of possible causes of heterogeneity.	Sections 3.4 and 3.13; Tables 2 and 5.	**Reported**	Informal heterogeneity patterns were reported across species, interventions, thermal protocols, durations, outcome domains and methodological quality.
**20d**	Results	Synthesis results: sensitivity analyses	Present results of all sensitivity analyses conducted.	Not applicable.	**N/A**	No sensitivity analyses were conducted.
**21**	Results	Reporting biases	Present assessments of risk of bias due to missing results for each synthesis assessed.	Section 2.8; Section 4.9.	**N/A**	No formal assessment of reporting bias due to missing results was performed; potential language and publication bias were discussed as limitations.
**22**	Results	Certainty of evidence	Present certainty or confidence assessments for each outcome or synthesis assessed.	Section 2.9; Table 1; Section 3.13.	**Reported**	Class-level qualitative confidence was reported, but confidence was not presented separately for every major outcome-domain synthesis and the assessor process was not stated.
**23a**	Discussion	Interpretation	Provide a general interpretation of the results in the context of other evidence.	Sections 4.1–4.8; Conclusions.	**Reported**	Findings were interpreted mechanistically and translationally while being framed as evidence-map support rather than universal formulation guidance.
**23b**	Discussion	Limitations of evidence	Discuss limitations of the evidence included in the review.	Sections 3.12, 3.13 and 4.9.	**Reported**	The manuscript discussed species imbalance, heterogeneous thermal protocols, experimental-unit concerns, incomplete reporting and limited recovery, disease-challenge and omics evidence.
**23c**	Discussion	Limitations of review processes	Discuss limitations of the review processes used.	Section 4.9.	**Reported**	Review-process limitations included English-only peer-reviewed eligibility, no systematic gray-literature search, no expert solicitation, possible publication bias and inability to pool heterogeneous evidence.
**23d**	Discussion	Implications	Discuss implications for practice, policy and future research.	Sections 4.8 and 4.10; Conclusions; Table 6; Figure 10.	**Reported**	Practical feeding phases, methodological priorities and future research directions were provided.
**24a**	Other	Registration	Provide registration information, including the register name and registration number, or state that the review was not registered.	Section 2.1; OSF registration DOI.	**Reported**	Retrospective OSF registration was reported with registration ID q5csy and DOI 10.17605/OSF.IO/PVG4H.
**24b**	Other	Protocol access	Indicate where the review protocol can be accessed, or state that a protocol was not prepared.	Section 2.1; OSF registration DOI.	**Reported**	Protocol information was stated to be accessible through the OSF registration DOI.
**24c**	Other	Protocol amendments	Describe and explain amendments to information provided at registration or in the protocol.	Section 2.1.	**Reported**	Retrospective registration was stated,
**25**	Other	Support	Describe financial or non-financial support and the role of funders or sponsors.	Funding; Author Contributions.	**Reported**	Funding sources were identified and the manuscript stated that the funders had no role in review design, screening, extraction, synthesis, interpretation or manuscript preparation.
**26**	Other	Competing interests	Declare competing interests of the review authors.	Conflicts of Interest.	**Reported**	The authors declared no conflicts of interest.
**27**	Other	Availability of data, code and materials	Report which materials are publicly available and where they can be found.	Supplementary Materials; Data Availability Statement; Supplementary File 1.	**Reported**	Supporting materials were listed.

Status definitions: Reported = all essential elements are identifiable; Partial = one or more essential elements are missing or not fully verifiable; N/A = the method or analysis was not conducted.

Reference: Page MJ, McKenzie JE, Bossuyt PM, et al. The PRISMA 2020 statement: an updated guideline for reporting systematic reviews. BMJ. 2021;372:n71. Checklist verified against the PRISMA 2020 main and expanded checklists.
